# A Systematic Review of Human Challenge Trials, Designs, and Safety

**DOI:** 10.1093/cid/ciac820

**Published:** 2022-10-11

**Authors:** Jupiter Adams-Phipps, Danny Toomey, Witold Więcek, Virginia Schmit, James Wilkinson, Keller Scholl, Euzebiusz Jamrozik, Joshua Osowicki, Meta Roestenberg, David Manheim

**Affiliations:** 1Day Sooner Research Team, Lewes, Delaware, USA; 1Day Sooner Research Team, Lewes, Delaware, USA; Warren Alpert Medical School of Brown University, Providence, Rhode Island, USA; 1Day Sooner Research Team, Lewes, Delaware, USA; 1Day Sooner Research Team, Lewes, Delaware, USA; 1Day Sooner Research Team, Lewes, Delaware, USA; RAND Corporation, Pardee RAND Graduate School, Santa Monica, California, USA; The Ethox Centre & Wellcome Centre for Ethics and the Humanities, Nuffield Department of Population Health, University of Oxford, Oxford, United Kingdom; Monash Bioethics Centre, Monash University, Clayton, VIC, Australia; Royal Melbourne Hospital Department of Medicine, University of Melbourne, Parkville, VIC, Australia; Murdoch Children's Research Institute, Parkville, VIC, Australia; Infectious Diseases Unit, Department of General Medicine, Royal Children's Hospital Melbourne, Parkville, VIC, Australia; Department of Parasitology, Leiden University Medical Centre, Leiden, The Netherlands; Department of Infectious Diseases, Leiden University Medical Center, Leiden, ZAThe Netherlands; 1Day Sooner Research Team, Lewes, Delaware, USA; Technion, Israel Institute of Technology, Haifa, Israel; ALTER, Association for Long Term Existence and Resilience, Rehovot, Israel

**Keywords:** systematic review, human challenge trial, controlled human infection model, risk mitigation, adverse events

## Abstract

**Background:**

Few studies have assessed participant safety in human challenge trials (HCTs). Key questions regarding HCTs include how risky such trials have been, how often adverse events (AEs) and serious adverse events (SAEs) occur, and whether risk mitigation measures have been effective.

**Methods:**

A systematic search of PubMed and PubMed Central for articles reporting on results of HCTs published between 1980 and 2021 was performed and completed by 7 October 2021.

**Results:**

Of 2838 articles screened, 276 were reviewed in full. A total of 15 046 challenged participants were described in 308 studies that met inclusion criteria; 286 (92.9%) of these studies reported mitigation measures used to minimize risk to the challenge population. Among 187 studies that reported on SAEs, 0.2% of participants experienced at least 1 challenge-related SAE. Among 94 studies that graded AEs by severity, challenge-related AEs graded “severe” were reported by between 5.6% and 15.8% of participants. AE data were provided as a range to account for unclear reporting. Eighty percent of studies published after 2010 were registered in a trials database.

**Conclusions:**

HCTs are increasingly common and used for an expanding list of diseases. Although AEs occur, severe AEs and SAEs are rare. Reporting has improved over time, though not all papers provide a comprehensive report of relevant health impacts. We found very few severe symptoms or SAEs in studies that reported them, but many HCTs did not report relevant safety data. This study was preregistered on PROSPERO as CRD42021247218.

## Teaser

A systematic review of health risks in human challenge trials published between 1980 and 2021 found no reported deaths or cases of permanent damage. Of challenged participants, 0.2% experienced serious adverse events and no deaths were observed.

Human challenge trials (HCTs) are a clinical research method in which volunteers are exposed to a pathogen to derive scientifically useful information about the pathogen and/or an intervention [1]. Such trials have been conducted with ethical oversight since the development of the modern institutional review system of clinical trials in the 1970s. More recently, there has been renewed discussion about the ethical and practical aspects of conducting HCTs, largely fueled by interest in conducting HCTs for severe acute respiratory syndrome coronavirus 2. Past reviews of HCTs focused on reporting methods [2] and safety for single pathogens [3–6], but these did not explicitly evaluate the safety of HCTs by assessing reported adverse events (AEs) and serious adverse events (SAEs) across a range of pathogens. Furthermore, many additional HCTs have been performed since the publication of these reviews. To better inform discussions about future uses of HCTs, including during pandemic response, this article presents a systematic review of challenge trials since 1980 and reports on their clinical outcomes, with particular focus on risk of AEs and risk mitigation strategies.

HCTs are often used to support development of therapies and vaccines more efficiently than conventional clinical trials [6, 7] and have recently been discussed as particularly valuable in the context of novel disease pandemics such as coronavirus disease 2019, Zika virus, or a future disease X [8, 9]. The benefits of such trials include defining and evaluating correlates of protection [10]; the first Food and Drug Administration (FDA)-approved cholera vaccine, Vaxchora, which proved its efficacy using a small HCT [7]; a contribution to the development of the FDA-approved therapeutic oseltamivir for influenza [11]; the Vi-tetanus toxoid conjugate vaccine for *Salmonella typhi* [12]; and dosing schedules and adjuvant selection for the RTS,S/AS01 malaria vaccine [13, 14].

Arguments against the use of HCTs have centered around ethics of participant compensation and the populations represented, and whether the risks and lack of personal benefit can be compatible with the principle of *primum non nocere* [15, 16] because of the potential risks they may inflict on a study population. Despite the debate, there is a long-standing consensus that infecting healthy volunteers is ethically justifiable as long as the risk of harm is acceptably low [15]. HCTs can therefore be ethical, based on a case-by-case assessment of risk as part of wider research ethics oversight mechanisms.

AEs related to challenge are 1 measure of health risk in HCTs. AEs refer to “any untoward medical occurrence associated with the use of a drug in humans” [17]. The FDA considers challenge agents as investigational new drugs [18], such that AEs in HCTs refer to any untoward medical occurrence associated with the challenge. AEs that result in death, hospitalization, disability, permanent damage, or other important medical events are reported as SAEs [17]. AEs graded “severe” by studies are distinct from SAEs in most cases, usually because they are not life-threatening or do not require hospitalization.

A systematic review was performed to characterize the frequency and nature of AEs and SAEs in HCTs related to the challenge and the risk mitigation measures used. The review also investigated the pathogens studied, the clinical outcomes in participants, study registration in databases, the number and uses of HCTs over time, and the quality of data reporting.

## METHODS

### Search Strategy

A systematic review of records from 1980 to 2021 indexed in the PubMed and PubMed Central databases was performed to identify published articles describing HCTs. Articles published before 1980 were not assessed because the modern institutional review system was not in place until after the 1979 Belmont report. The initial search was preregistered on PROSPERO as CRD42021247218 [19], but it identified few studies published before 2010. Additional searches were performed to appropriately discover studies for each decade of interest, as detailed in the amended preregistration [19] and the Supplementary Methods. The database search strategy is presented in Table 1. Further manual searches of references lists and reviews were performed to identify additional articles describing HCTs that were missed.

**Table 1.** Search Strategy

Search Number	Search Purpose	Database Accessed	Date Accessed	Query Text	Results, n
Search 1	Articles from all decades	PMC	20 April 2021	(((((“human challenge”) OR (“controlled human infection”)) AND (trial OR vaccine OR model)) AND (((“adverse events”) OR (medical* AND “significant event” OR “significant events”)))) AND (“1980”[PMC Live Date] : “2021/04/20”[PMC Live Date]))	417
Search 2	Articles before 1990	PubMed	6 January 2021	((“human challenge”) OR (“controlled human infection”) OR (“experimental” AND “infection” AND “human*”) OR (“wild-type virus” AND infection)) AND (trial OR vaccine OR model OR inoculat*) AND ((“adverse events”) OR (medical* AND “significant event” OR “significant events”) OR (illness)) AND (0:1990[pdat])	90
Search 3	Articles between 1990 and 2000	PubMed	6 January 2021	((experimental* AND infect*) OR (“wild-type” AND inoculat*) OR (volunteer* AND inoculat*)) AND (trial OR vaccine OR model OR inoculat* OR stud*) AND (“adverse events” OR (medical* AND “significant event*”) OR “illness”) AND (1990:2000[pdat])	326
Search 4	Articles between 2000 and 2010	PubMed	6 January 2021	((experimental* AND infect*) OR (“wild-type” AND inoculat*) OR (volunteer* AND inoculat*)) AND (trial OR vaccine OR model) AND (“adverse events” OR (medical* AND “significant event*”) OR “illness”) AND (2000:2010[pdat])	483
Search 5	Articles that were otherwise missed	PubMed	10 July 2021	((human challenge AND trial) OR (human challenge AND vaccine) OR (controlled AND human AND infection AND model)) AND (severe AND events) AND (1980:2021[pdat])	1338

Abbreviation: PMC, PubMed Central.

### Screening Process

Titles and abstracts of search results were manually screened by 3 authors working independently to identify articles that were eligible for full-text review. Case reports, reviews, articles not available in English, studies that did not meet the criteria for an HCT, and articles published before 1980 were excluded. Secondary reviews of 2 past reviews [2, 20] were also performed to identify more articles that were missed by the searches. Articles that described studies that performed secondary analysis of results from previously conducted HCTs were excluded, but their reference lists were reviewed to identify the original publication of these results.

### Full-text Review Process

The unit of analysis is the individual study, as described within a published article detailing results. Individual studies were identified by trial registration. If trial registration was not reported, studies were counted per the article description, or as a single study if participants were challenged with a single pathogen. If multiple articles were published discussing the same study, the earliest published article was included. In some cases, multiple articles were combined (see Supplementary Methods).

There is an ongoing discussion on the precise definition of an HCT [21]. In general, studies that had been completed and involved intentional exposure of human volunteers to a pathogen were included. Challenges with candidate vaccine viruses were also included, as were studies in which previously challenged participants were challenged again with the same pathogen. Consistent with Kalil et al, studies involving live, attenuated vaccines that were not followed by intentional infection, as well as data from phases of studies involving immunization or vaccination with live, attenuated vaccines or other methods that could have potentially resulted in infection, but that are not generally referred to as HCTs, were excluded [22].

### Data Collection Process

At least 2 reviewers independently examined each publication selected for full-text review and any discrepancies were either reconciled or resolved by the senior author. Data collection was performed manually and results were input into a spreadsheet.

### Data Extraction

The following numerical data were extracted from each study: year of article publication, size of cohort, sex breakdowns; mean or median age, standard deviation, and age range; number of participants challenged, number of challenged participants infected with pathogen, number of participants in control group (those who did not undergo a challenge), number of control participants infected with pathogen, number of control participants with at least 1 AE, and number of challenged participants with: (1) at least 1 AE, (b) at least 1 “severe” or “very severe” (grade 3 or higher) AE, (3) at least 1 SAE.

In addition, the following nonnumerical data were extracted from each study: clinical trial registration, pathogen assessed, definition of infection, definition of AEs, treatments administered to participants, risk mitigations taken, ethics committee and review board approvals reported, and a brief description of the study design.

For articles that reported separate study arms that were all exposed to a pathogen within a single pathogen category, data were summed across all arms to be treated as a single study. Data from rechallenges were extracted separately and treated as individual studies. No treatment effect measures were extracted.

AEs among challenged participants that were not related to challenge (such as AEs related to vaccination or drug treatment) were not extracted (see Supplementary Methods). For studies that did not define and/or report AEs, reported symptom data were extracted instead. For studies that did not define and/or report SAEs, reported symptom data that met the 2016 definition of SAEs provided by the FDA [17] based on reviewer judgment were extracted as SAEs.

## RESULTS

### Study Selection

**Figure 1.**
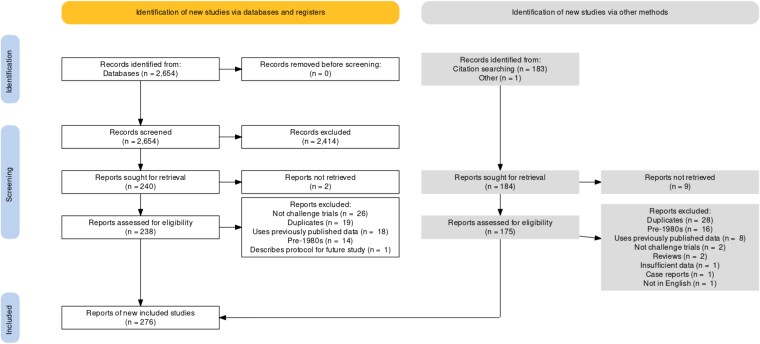
Preferred Reporting Items for Systematic Reviews and Meta-Analyses flow diagram.

Figure 1 shows a Preferred Reporting Items for Systematic Reviews and Meta-Analyses flowchart of study selection. Searches yielded a total of 2654 results; 183 additional results were added by citation searching the reference lists of 2 past reviews [2, 20] and articles identified among search results that used data from prior HCTs. One article [23] provided updated data for another [24]. Eleven results were not retrieved (5 with no full text available and 6 with unpublished data) and 47 duplicates were removed. No further efforts were made to identify unpublished or unidentified work. A total of 276 articles were included, describing 308 studies from which data were extracted. Excluded results were primarily reviews and articles discussing non-HCT clinical trials. See the Supplementary references for the complete reference list of included articles.

### Results of Individual Studies

Data from 284 studies, with 14 628 challenged participants, were extracted (Table 2). Additional data were extracted from 24 rechallenge studies (Supplementary Tables 3, 4, 5, and 8). Between 9917 and 10 277 challenged participants (67.8%-70.3%) were diagnosed with infection. The dataset and code used for generating all results and tables are publicly available [26].

**Table 2.** Number of Studies, Number of Participants, and Number of Infections in Published HCTs by Decade

Decade	Studies, n	Participants Challenged, n	Control Participants, n	Challenged Participants Diagnosed With Infection^a^, n
1980s	31	1761	18	1272-1385
1990s	68	4181	47	2956-3040
2000s	57	2907	37	2172-2193
2010s	106	4789	256	2860-2980
2020s	22	990	75	657-679^b^
Total	284	14 628	433	9917-10 277^b^

Abbreviation: HCT, human challenge trial.

A range of values is given to account for unclear data reporting by some studies.

One additional control (nonchallenged) participant was diagnosed with infection with influenza in a human challenge-transmission model [25].

### Reported AEs and Unreported Data

**Figure 2.**
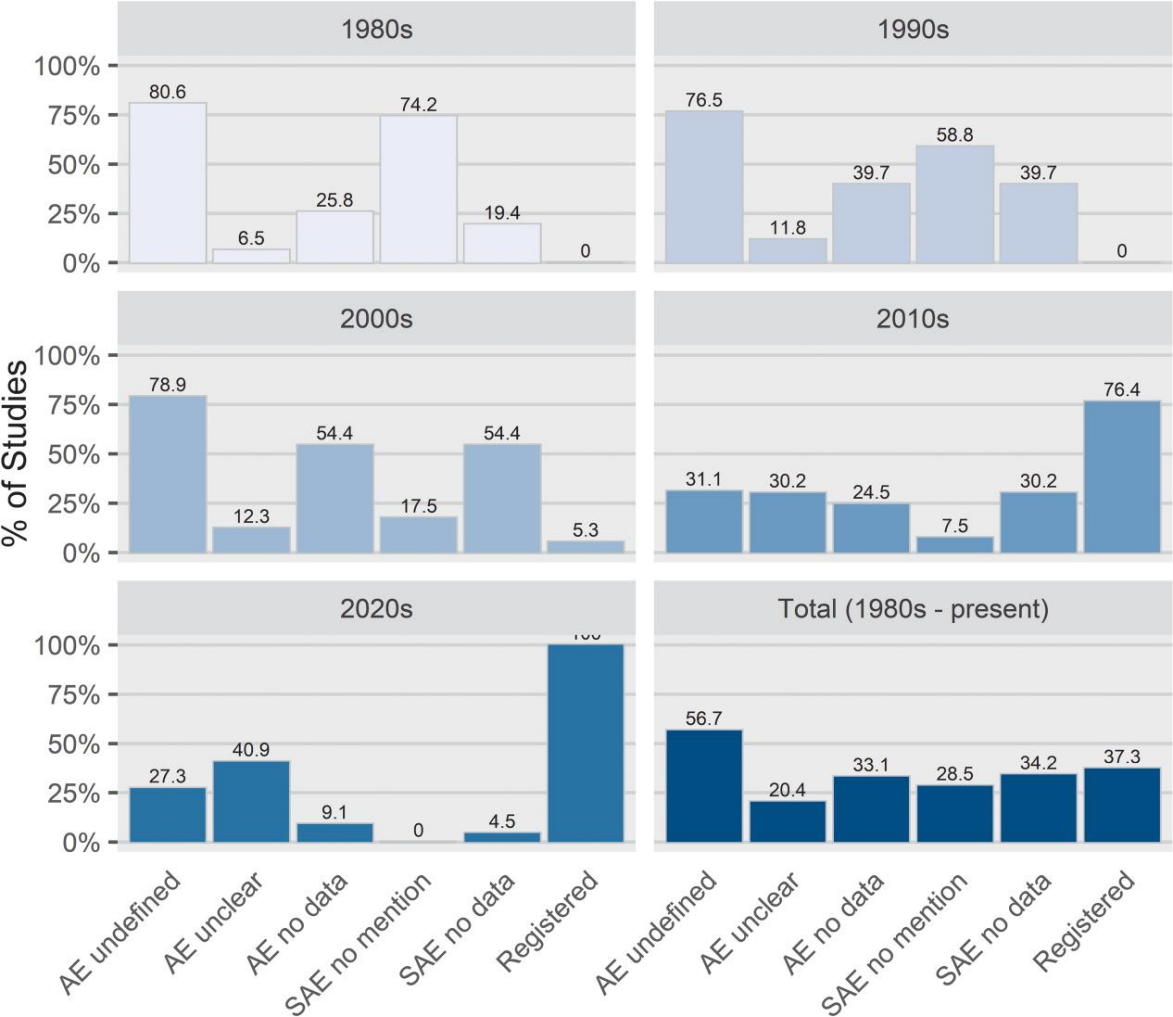
Reporting and database registration in published human challenge trials.

Among 284 studies, 94 and 97 did not report any AE or SAE data, respectively (Table 3, Figure 2). The precise number of participants experiencing at least 1 SAE could not be extracted from 2 studies: 1 lost challenged subjects' records in a flooded storage facility [27] and the other did not provide any detail on the AEs observed [28].

**Table 3.** Data Reporting and Database Registration in Published HCTs by Decade

Decade	Studies, n	Studies That Do Not Define AEs, n (%)	Studies With Unclear AE Data, n (%)	Studies With No AE Data, n (%)	Studies That Do Not Mention SAEs, n (%)	Studies With No SAE Data, n (%)
1980s	31	25 (80.6)	2 (6.5)	8 (25.8)	23 (74.2)	6 (19.4)
1990s	68	52 (76.5)	8 (11.8)	27 (39.7)	40 (58.8)	27 (39.7)
2000s	57	45 (78.9)	7 (12.3)	31 (54.4)	10 (17.5)	31 (54.4)
2010s	106	33 (31.1)	32 (30.2)	26 (24.5)	8 (7.5)	32 (30.2)
2020s	22	6 (27.3)	9 (40.9)	2 (9.1)	0 (0.0)	1 (4.5)
Total	284	161 (56.7)	58 (20.4)	94 (33.1)	81 (28.5)	97 (34.2)

Abbreviations: AE, adverse event; HCT, human challenge trial; SAE, serious adverse effect.

Among 10 325 challenged participants in studies that reported AEs, between 4317 (41.8%) and 5730 (55.5%) experienced at least 1 AE (Table 4). Among 5083 challenged participants in studies that graded severity of AEs, between 285 (5.6%) and 801 (15.8%) experienced at least 1 severe or very severe (grade 3 or higher) AE (Table 5). The range in possible AE values is greater in more recent decades as a result of more studies reporting AEs by individual or symptom, rather than reporting the total number of participants with at least 1 AE. Nineteen studies included control (nonchallenged) participants (n = 433); only 2 of these studies reported AE data for control participants (n = 69). Between 7 (10.1%) and 12 (17.4%) control participants experienced at least 1 AE.

**Table 4.** AEs in Published HCTs by Decade

Decade	Studies^a^, n	Participants Challenged, n	Challenged Participants With AEs (minimum^b^), n (%)	Challenged Participants With AEs (maximum^b^), n (%)
1980s	23	1448	389 (26.9)	428 (29.6)
1990s	41	2875	1192 (41.5)	1384 (48.1)
2000s	26	1984	743 (37.4)	1001 (50.5)
2010s	80	3139	1576 (50.2)	2210 (70.4)
2020s	20	879	417 (47.4)	707 (80.4)
Total	190	10 325	4317 (41.8)	5730 (55.5)

Abbreviations: AE, adverse event; HCT, human challenge trial.

94 studies that did not report AE data are excluded, see Supplementary Data, Tables 1 and 2.

Minimum and maximum values are given to account for unclear data reporting by some studies.

**Table 5.** Severe AEs in Published HCTs by Decade

Decade	Studies^a^, n	Participants Challenged, n	Challenged Participants With Severe or Very Severe (≥Grade 3) AEs (Minimum^b^), n (%)	Challenged Participants With Severe or Very Severe (≥Grade 3) AEs (Maximum^b^), n (%)
1980s	3	77	9 (11.7)	25 (32.5)
1990s	8	429	23 (5.4)	23 (5.4)
2000s	12	1984	31 (1.6)	102 (5.1)
2010s	57	1954	179 (9.2)	473 (24.2)
2020s	14	639	43 (6.7)	178 (27.9)
Total	94	5083	285 (5.6)	801 (15.8)

Abbreviations: AE, adverse event; HCT, human challenge trial.

190 studies that did not report severe AE data are excluded, see Supplementary Data, Tables 1 and 2.

Minimum and maximum values are given to account for unclear data reporting by some studies.

Among 10 016 challenged participants in studies that reported SAEs, 23 (0.2%) experienced at least 1 SAE (Table 6). Among 146 rechallenged participants in studies that reported SAEs, 1 additional participant (0.7%) experienced at least 1 SAE (Supplementary Table 6). No fatalities were reported. SAEs are described in more detail in Table 7, and some SAEs deemed not related to challenge are discussed further in Supplementary Table 7.

**Table 6.** Serious AEs in Published HCTs by Decade

Decade	Studies^a^, n	Participants Challenged, n	Challenged Participants With SAEs, n (%)
1980s	25	1469	6 (0.4)
1990s	41	2799	1 (0.0)
2000s	26	1623	1 (0.1)
2010s	74	3194	13 (0.4)
2020s	21	931	2 (0.2)
Total	187	10 016	23^b^ (0.2)

Abbreviations: AE, adverse event; HCT, human challenge trial; SAE, serious adverse event.

97 studies that did not report SAE data are excluded, see Supplementary Data, Tables 1 and 2.

One additional SAE from a rechallenge is described in Table 7 but not included in this total.

**Table 7.** Descriptions of SAEs in Published HCTs by Pathogen Category

Pathogen Category	Participants With ≥1 SAE, n	Description	Outcomes	Long-term Follow-up	Dataset File Name	Supplementary Reference Numbers^a^
*Escherichia coli*						
…	2	Clinical relapse of diarrhea and vomiting with trimethoprim-resistant strain isolated in stools, after initial improvement following trimethoprim treatment.	ND	ND	Black 1982	27
…	4	“…four subjects became sufficiently ill that they received adjunctive therapy,” including intravenous fluids, antiemetics, or oral antibiotics.	ND	ND	Graham 1983	100
Influenza viruses						
…	1	A 21-y-old male developed dilated cardiomyopathy, possibly related to experimental influenza B infection.	Resolved with ACE-I treatment.	Clinically stable with low-normal cardiac output on echocardiography after ∼5 y.	Barroso 2005	18
Norovirus						
…	4	Severe vomiting and/or diarrhea.	ND	No further SAE reported over 12 mo.	Bernstein 2015	22
*Plasmodium* spp						…
…	1	Probable case of acute myocarditis 12 d after challenge and 1 d after diagnosis and treatment with atovaquone/proguanil for malaria. Definite etiology and mechanism have not been established.	Clinical and biochemical recovery within ∼2 wk.	Normal cardiac MRI after ∼5 mo, edema resolved, with decreased but persistently delayed enhancement of subepicardial and mid-wall regions.	Bastiaens 2016	19
…	1	Asymptomatic molecular relapse with unexpected positive qPCR on day 28 (smear negative) after treatment with atovaquone/proguanil.	Remained asymptomatic. Single further borderline positive qPCR. Repeated negative smears. Retreated with chloroquine. Smear results and qPCR subsequently negative.	*Plasmodium falciparum* culture of blood from day 28 was negative after 4 wk incubation.	Lyke 2015	165
…	3	Hepatitis temporally related and considered as likely attributable to ferroquine treatment.	ND	ND	McCarthy 2016	175
…	1	Overnight hospital admission for treatment with acetaminophen and chloroquine. Mild transient thrombocytopenia, leukopenia, pyuria, hematuria.	ND	ND	Rickman 1990	209
…	1	Chest pain 1 d after treatment initiated with atovaquone/proguanil, initially considered as possibly consistent with angina pectoris.	Spontaneous resolution of pain within 1 h. Brief admission for cardiac monitoring. Single abnormal ECG (negative T-wave in V2) reverting to baseline. Normal serial troponin levels.	ND	Roestenberg 2013	210
Respiratory syncytial virus						
…	1	Acute myocarditis.	ND	ND	DeVincenzo 2020	68
Salmonella spp.						
…	1	Persistent nausea, vomiting, tachycardia, not improved by oral antiemetic treatment.	Overnight admission for intravenous fluid and ceftriaxone. Discharged to complete oral ciprofloxacin course.	ND	Gibani 2020	95
…	1	Elevated alanine aminotransferase (898 IU/L) 5 d after diagnosis, ascribed to paratyphoid fever plus possible adverse drug reaction.	Complete biochemical recovery. Further acetaminophen withheld and azithromycin switched to ciprofloxacin.	ND	Gibani 2020	95
…	1	Reactive arthritis possibly related to challenge or antibiotic treatment.	ND	ND	Jin 2017	143
*Shigella* spp						
…	2	Two subjects with asymptomatic hyperbilirubinemia at day 14 visit.	Total bilirubin levels returned to normal by day 28, without treatment.	No concerns at day 42 telephone assessment.	Bodhidatta 2012	30

Abbreviations: ACE-I, angiotensin converting enzyme inhibitor; ECG, electrocardiogram; HCT, human challenge trials; MRI, magnetic resonance imaging; ND, no data; qPCR, quantitative polymerase chain reaction SAE, serious adverse event.

These numbers refer to the reference number of each study in the Supplementary reference list in the Supplementary Materials.

### Studies by Pathogen

**Figure 3.**
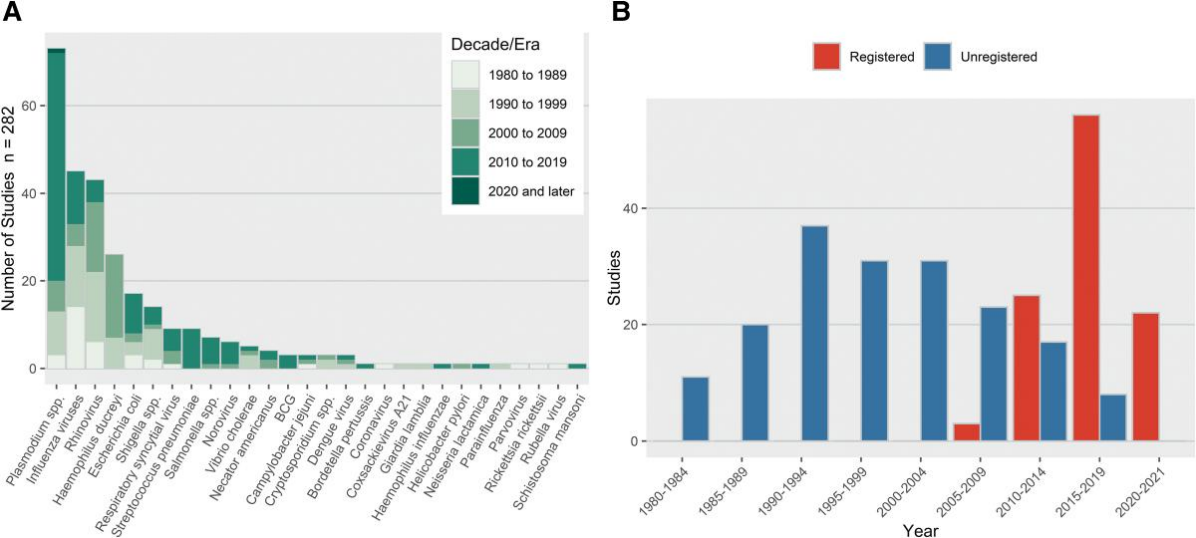
(*A*) Studies by pathogen. (*B*) Data reporting and database registration in published human challenge trials.

The numbers of studies and participants challenged within each category of pathogen are presented in Table 8, and Figure 3*A* illustrates studies of different pathogens have occurred over time. There were 28 pathogen categories, with the most commonly studied being *Plasmodium* spp (73 studies, 1689 participants), influenza viruses (45 studies, 3536 participants), and rhinovirus (43 studies, 4332 participants). Studies investigating *Plasmodium* spp had the greatest number of challenged participants with SAEs, with 7 SAEs (of 23 in all nonrechallenge studies) occurring among 1129 participants in 52 studies. Studies investigating norovirus had the greatest proportion of SAEs to number challenged, with 4 SAEs occurring among 163 participants in 3 studies.

**Table 8.** Number of Published HCTs, Number of Participants, Number Infected, and Number With SAEs by Pathogen Category

Pathogen Category	Studies, n	Participants Challenged Across All Studies, *n*	Challenged Participants Diagnosed With Infection Across All Studies, n (%)	Studies That Reported SAEs, n	Participants Challenged Across Studies That Reported SAEs, n	Challenged Participants With SAEs Across Studies That Reported SAEs, n (%)
BCG	3	128	88 (68.8)	3	128	0 (0.0)
*Bordetella pertussis*	1	34	19 (55.9)	1	34	0 (0.0)
*Campylobacter jejuni*	3	197	178 (90.4)	3	197	0 (0.0)
Coronavirus	1	55	50 (90.9)	1	55	0 (0.0)
Coxsackievirus A21	1	31	29 (93.5)	1	31	0 (0.0)
*Cryptosporidium* spp	3	79	45 (57.0)	3	79	0 (0.0)
Dengue virus	3	104	70 (67.3)	2	63	0 (0.0)
*Escherichia coli*	17	559	395 (70.7)	9	300	6 (2.0)
*Giardia lamblia*	1	19	5 (26.3)	0	0	0 (0.0)
*Haemophilus ducreyi*	26	218	180 (82.6)	0	0	0 (0.0)
*Haemophilus influenzae*	1	15	9 (60.0)	0	0	0 (0.0)
*Helicobacter pylori*	1	20	18 (90.0)	1	20	0 (0.0)
Influenza viruses	45	3536	2224 (62.9)	38	3011	1 (0.0)
*Necator americanus*	4	69	44 (63.8)	2	45	0 (0.0)
*Neisseria lactamica*	1	292	97 (33.2)	1	292	0 (0.0)
Norovirus	6	293	150 (51.2)	3	163	4 (2.5)
Parainfluenza	1	83	34 (41.0)	1	83	0 (0.0)
Parvovirus	1	9	5 (55.6)	1	9	0 (0.0)
*Plasmodium* spp	73	1689	1313 (77.7)	52	1129	7 (0.6)
Respiratory syncytial virus	9	502	332 (66.1)	7	420	1 (0.2)
Rhinovirus	43	4332	3285 (75.8)	31	2560	0 (0.0)
*Rickettsia rickettsii*	1	22	18 (81.8)	1	22	0 (0.0)
Rubella virus	2	40	28 (70.0)	2	40	0 (0.0)
*Salmonella* spp	7	374	197 (52.7)	6	282	2 (0.7)
*Schistosoma mansoni*	1	17	17 (100.0)	1	17	0 (0.0)
*Shigella* spp	14	708	386 (54.5)	10	445	2 (0.4)
*Streptococcus pneumoniae*	10	936	330 (35.3)	5	530	0 (0.0)
*Vibrio cholerae*	5	267	199 (74.5)	2	61	0 (0.0)
Total	284	14 628	9745 (66.6)	187	10 016	23 (0.2)

Abbreviations: HCT, human challenge trial; SAE, serious adverse event.

### Reporting AEs and Use of Trial Registries Over Time

Overall, the number of challenge studies has been increasing each decade (Figure 3*B*). Before the 2000s, many studies did not report AEs, but instead reported comparable symptom data. These were extracted as AEs. Of the 283 included studies, 123 explicitly mentioned or defined AEs, but not all reported them for the challenge phase specifically. The proportion of studies with definitions has increased over time, from only 19.4%, 23.9%, and 21.1% in the 1980s, 1990s, and 2000s, respectively, to 68.9% and 72.7% in the 2010s and 2020s (thus far), respectively. Results that exclude studies that did not explicitly mention AEs and SAEs are presented in Supplementary Tables 9 and 10.

The National Institutes of Health launched ClinicalTrials.gov on 29 February 2000. For National Institutes of Health-funded research after 2007, “applicable clinical trials” are required to be registered [29]. However, publication year lags year of registration, so it is unclear how much of the lack of registration is noncompliance and how much is delayed publication. Still, only 5.3% of included studies published in the 2000s were registered in at least 1 registry; 76.4% of included studies published in the 2010s were registered in at least 1 registry (Figure 2). Every included study published so far this decade was registered (Figure 2).

### Risk Mitigation

Text describing specific risk mitigation measures was found in 286 of the 308 studies, which is included in the dataset [26], and a descriptive summary follows. The qualitative nature of these mitigation descriptions precluded meaningful quantitative analysis.

Risk mitigation measures typically include evaluating participants' risk of disease if exposed to a challenge agent by using medical screening and assessing participants' medical histories. In some cases, checking for previous exposure to the pathogen was a risk mitigation strategy, but it could also be done for other reasons. Demographic criteria, pregnancy screening, assessment of cardiac risk, and assessment of weight and/or body mass index were often used to evaluate risk.

Some studies reported mitigation strategies for risks to nonparticipants, such as isolation throughout the duration of the study, requiring birth control, or excluding participants with employment posing risk of spread (for example, excluding food handlers in HCTs investigating *Escherichia coli*, norovirus, and *Salmonella* spp). Validity of informed consent was sometimes assessed by testing participants' understanding of the study protocol.

## DISCUSSION

The present review found a total of 24 (23 reported in traditional challenges, 1 in a rechallenge) SAEs and 0 reported deaths or cases of permanent damage among 15 046 participants in 308 studies spanning 1980 to 2021. It is unlikely that any SAEs captured in this review (Table 7) were life-threatening because the events were primarily brief hospitalizations for observation or supportive care requiring noninvasive interventions or falling under the broad category of “other serious (important medical events)” in the FDA definition of SAEs. The proportions of studies that define AEs and mention SAEs have increased over time, although inconsistent definitions make it challenging to compare reported data, particularly across studies investigating different pathogens. Unfortunately, the proportions of studies that do not report AE and SAE data related to challenges remained unacceptably high in the 2010s at 24.5% and 30.2%, respectively (Table 3). Although a high rate of failing to report SAEs may be indicative of their rarity in the HCT setting, clearer reporting would allow for better understanding of the risks and benefits of HCTs.

Issues surrounding AE reporting in clinical trials are not exclusive to HCTs [30]. However, confusion related to reporting challenge-related AEs is an issue specific to HCTs. For example, some studies identified “expected symptoms” as being distinct from AEs, only reported AEs related to interventions, or omitted discussion of AEs entirely. Additionally, clinical endpoints (such as moderate to severe diarrhea in *E coli* HCTs) were not always reported as AEs by the study. There is a greater degree of consistency for SAE reporting generally in agreement with the FDA definition [17], but many studies, especially those published before 2000, did not define or report SAEs. Guidelines for HCT reporting have been suggested [22] but have not yet been adopted. Accordingly, a major conclusion of this review is that in addition to a greater effort to standardize AE reporting in general, which others have postulated [30], these standardization efforts are particularly valuable to HCTs.

The number of new HCTs has been increasing; however, it is unclear whether this increase is proportional to the general growth trend in the number of new (non-HCT) clinical trials. Since 2010, pathogens such as *Bordetella pertussis*, *Schistosoma mansoni*, and *Streptococcus pneumoniae* have been studied in HCTs for the first time. Figure 3*A* shows that the number of influenza and rhinovirus HCTs has declined somewhat over time, following the discontinuation of several research programs focused on common cold, whereas the number of *Plasmodium* spp HCTs sharply increased in the 2010s. These trends demonstrate that HCTs are an increasingly ubiquitous tool and that their relative speed allows researchers to investigate new pathogens of interest more rapidly than in traditional clinical trials.

Limitations of this review are primarily related to uncertainties around the accuracy of AE reporting. This includes potential bias in AE reporting, inconsistent reporting, and difficulty in precisely estimating the rates of events based on provided data. Many studies reported either no or unclear AE and/or SAE data, and issues of censoring and misclassification are common with respect to AE reporting in general [31]. To partially address issues with different standards for reporting over time, we extracted symptom data as AE and/or SAE data from studies that did not mention or define AEs/SAEs, but this means that AEs for decades in which these studies occurred are not fully comparable. The review is further limited by our inability to locate some results, including published HCTs that were not on PubMed [32] and HCTs whose results have only been published as case reports [33]. These limitations further highlight the need for improvements in the field of HCTs with respect to AE reporting and availability of results. Future work building off of this review includes policy recommendations around the issues of standardization and AE reporting, investigating the registration of HCTs in databases, and further qualitative analysis of risk mitigation measures in published articles.

## CONCLUSIONS

The recent literature contains hundreds of HCTs involving more than 10 000 participants and only 24 SAEs. With the qualification that systematic AE reporting in many studies has been incomplete, reports of severe symptoms and SAEs related to infectious challenge in HCTs are notably infrequent. Specifically, participation in an HCT has not been associated with permanent impairment or death. HCTs are now routinely used to understand infectious dose, disease progression, clinical efficacy of novel interventions, and immune response for a wide variety of pathogens. As evidenced by recent HCTs for coronavirus disease 2019, they may be conducted for novel as well as familiar diseases. This review can help support public discussion and expert deliberation regarding the safety of HCTs. It may also inform future discussions among HCT researchers and members of ethics review committees regarding the planning, conduct, and reporting of future HCTs.

Preregistration, Protocol, and Conflict of Interest Disclosures

The review was preregistered on PROSPERO as CRD42021247218, risk outcomes and risk mitigation measures in human challenge trials: a systematic review. As mentioned previously, the preregistration was amended to include additional searches and data. The review protocol is available online as Supplementary Material—Protocol.

## Supplementary Data

Supplementary materials are available at *Clinical Infectious Diseases* online. Consisting of data provided by the authors to benefit the reader, the posted materials are not copyedited and are the sole responsibility of the authors, so questions or comments should be addressed to the corresponding author.

## Notes

*Author contributions*. D. M., W. W., and V. S. conceived of the idea. E. J., J. O., M. R., and W. W. provided initial feedback and refined the idea. D. T. and D. M. designed and preregistered the systematic review, with feedback and expert guidance from E. J., J. O., and M. R. D. T. led the initial review for inclusion, with J. A. P. and K. S. The full-text reviews were done by J. A. P., D. T., and 2 nonauthor reviewers thanked in the Acknowledgments: S. K. and D. K. Disputes were resolved by D. M. Guidance on inclusion criteria and interpretation was provided by E. J., K. S., and J. O. J. A. P. and D. T. led writing of the manuscript, with supervision and assistance by D. M., V. S., W. W., K. S., E. J., and J. O. J. W. managed the dataset, performed analysis, led creation of visualizations, and produced tables and data summaries. D. M. and W. W. contributed to the visualizations.

***Acknowledgments***. Thank you to 1DaySooner for supporting this work, and to the 1DaySooner Scientific Advisory board, which includes coauthors D. M., V. S., and W. W., for reviewing the proposed study. The authors thank Steffen Kamenicek and Daniel Kaufman, who assisted with full-text review of papers and data extraction. They also thank Steffen Kamenicek and River Bellamy for additional assistance with reviewing the text, tables, and figures before submission.

***Financial support***. This work was supported by 1Day Sooner (D. T., J. A. P., K. S., W. W.). D. M. was supported by grants from the Center for Effective Altruism’s Long Term Future Fund and Guarding Against Pandemics (materials, Article Processing Charges). E. J.'s work was supported by the Wellcome Trust, including current grants 221 719 and 216 355. J. O. reports support from Melbourne Children's campus (Royal Children's Hospital, University of Melbourne, Murdoch Children's Research Institute) and the Australian National Health and Medical Research Council (NHMRC). During manuscript preparation/submission, V. S. reports a position as the director of research for 1Day Sooner, which funded the time spent working on the manuscript.

***Availability of data, code, and other materials*.** The complete dataset of included studies is publicly available online [26].

## Supplementary Material

ciac820_Supplementary_Data
