# Supplementary material for: A Systematic Review of Human Challenge Trials, Designs, and Safety
Source: Clin Infect Dis. 2022 Oct 11;76(4):609–19. doi: 10.1093/cid/ciac820 (PMC9938741; doi:10.1093/cid/ciac820)
Supplement: ciac820_Supplementary_Data [file ciac820_supplementary_data.zip › CID 5.31.22 - Supplementary Materials - Resubmission.docx]

**A Systematic Review of Human Challenge Trials, Designs, and Safety**

Supplementary Materials

Jupiter Adams-Phipps*, Danny Toomey*, Witold Więcek, Virginia Schmit, James Wilkinson, Keller Scholl, Euzebiusz Jamrozik, Joshua Osowicki, Meta Roestenberg, David Manheim^^[[1]](#footnote-0)^^

*Equal Contribution

[**Supplementary Methods**](#_bnwam8tvmw0) **3**

[Search Strategy Rationale](#_t73o58lbix69) 3

[Adverse Events](#_9ym7p85qyc7b) 3

[Counting Studies](#_y479kom04g33) 4

[Combined Data](#_g7iqvqi1dt8y) 5

[Risk of Bias](#_knux5n3n8370) 5

[Additional Safety Concerns](#_7ppo87pfsslv) 5

[**PRISMA Checklists**](#_5lifvo6xjro1) **7**

[PRISMA Checklist](#_9c2dzg19ug4d) 7

[PRISMA 2020 for Abstracts Checklist](#_y18o4hm6hvzh) 9

[**Supplementary Tables**](#_g92yec1118z) **11**

[Data Categorization](#_tm7ypbbojjdj) 11

[Supplementary Table 1](#_pwj4zlkijkkx) 12

[Supplementary Table 2](#_cpv1dy7ddyjd) 13

[Supplementary Table 3](#_3e2eu6ye7l4r) 14

[Supplementary Table 4](#_7cmaezm4mbju) 15

[Supplementary Table 5](#_mgf3uhmui1gz) 16

[Supplementary Table 6](#_uqd5takyuolz) 17

[Supplementary Table 7](#_a2vyioi8rbdj) 18

[Supplementary Table 8](#_6zbyo3kzus6e) 21

[Supplementary Table 9](#_gargzqysu4rg) 22

[Supplementary Table 10](#_6gdy5dn3yg1s) 23

[**Supplementary References**](#_ac0ldbtyga9m) **24**

[Reference List of Included Studies](#_is0rx3ro6ub0) 24

[Reference List of Excluded Studies](#_19cazc88gv6) 59

# Supplementary Methods

## Search Strategy Rationale

Five separate searches were performed, as described in Table 1 in the paper. Search 1 was performed initially and subsequent searches 2-4 were performed to fill gaps left by search 1. After reviewing results, search 5 was performed to identify more papers that were otherwise missed. The search 1 algorithm was repeated in the PubMed and PubMed Central (PMC) databases, where it returned 24 and 417 results respectively. Because of this difference in hit rate, the PMC results were selected for screening. However, only 27 of these results were published before 2010, leaving our dataset skewed towards one decade. To address this skew, publications of known challenge trials that should have been included in our search results were used to identify terms that were standard usage in different decades. PubMed searches for MeSH terms in the title and abstract only, while PMC searches for MeSH terms throughout entire documents. As a result, PubMed results are pre-screened by title and abstract and usually have a lower hit count than PMC results. This is preferable in a systematic review workflow as long as PubMed’s pre-screening doesn’t produce too few results. Searches 2-4 cumulatively produced 899 results on PubMed and 17,593 results on PMC. Because 899 is an appropriate number of results to expect for this search, the PubMed results were screened instead of the PMC results. Likewise, search 5 was performed on PubMed, and produced 1,338 results.

## Adverse Events

This review investigates AEs as a measure of harm in order to evaluate the safety of HCTs. Accordingly, AEs that were not related to challenges (such as those reported related to vaccination or drug treatment) were not extracted. AEs related to challenge are additional risks on top of those that may be experienced in traditional clinical trials that do not involve intentional exposure to a challenge agent.

Many studies reported individual AEs by symptom or total individual AEs rather than the number of participants that experienced at least one AE. To handle this unclear AE data, the number of participants that must have experienced at least one AE based on the data that was provided was derived and extracted as a minimum value, and the lesser of the sum or the total number of participants challenged was extracted as a maximum value.

Additional assumptions regarding AEs were made for several studies:

- Schiff 2000: unclear whether AEs were treatment related or challenge related; counted as challenge related to avoid potential underestimate
- Sulyok 2017: unclear whether AEs were treatment related or challenge related; counted as challenge related to avoid potential underestimate
- Turner 1999: unclear whether AEs were treatment related or challenge related; counted as challenge related to avoid potential underestimate
- Turner 2001: unclear whether AEs were treatment related or challenge related; counted as challenge related to avoid potential underestimate
- Turner 2005: unclear whether AEs were treatment related or challenge related; counted as challenge related to avoid potential underestimate

## Counting Studies

As described in the paper, an individual “study” is identified by trial registration in a database (such as ClinicalTrials.gov), or per the article description if trial registration is not reported. Without universal trial registration, counting the number of “studies” based on what is reported in published articles is an inexact process. Articles often report multiple cohorts challenged at different times and with different strains within the pathogen being investigated, making it difficult to ascertain whether multiple “studies” occurred, or whether all challenges were part of a single “study.” It is understood that the number of studies described in the dataset used for this review may reasonably be counted differently by following different methodology; however, this does not impact data on challenged participants.

## Combined Data

Data from multiple articles that discussed the same study were combined in several instances:

- Rylance 2019 and Hales 2020 discussed the same study but reported slightly different data about it
- Lyke 2010 and Laurens 2013 had the same ClinicalTrials registration number, but each paper reported data for different cohorts
- Atmar 2008 did not contain updated data, which was found in Atmar 2014. Atmar 2014 was not found in searches, but was added to the review in order to properly extract data for the study identified in Atmar 2008

## Risk of Bias

Risk of bias in the conduct of individual studies was not assessed because the relevant aim of the review was to review reported data, and thereby assess whether data reporting in challenge trials has been sufficient.

## Additional Safety Concerns

A number of studies flagged safety concerns that could not be extracted quantitatively, which are reported here for the sake of completeness:

- Al-Tawfiq 1998: “Three volunteers were inoculated with a mixed culture of H. ducreyi and Pseudomonas aeruginosa”
- Feary 2009: “11 of the 13 participants who completed the study chose not to take the treatment, citing either a perceived improvement in hayfever symptoms, or that they wished to see if they had a change in symptoms the following year. In accordance with our protocol, participants in the placebo group were offered hookworm infection at the end of the trial, of whom 11 chose to be infected.”
- Fortney 2000: “Three volunteers were excluded from the analysis because they were inadvertently inoculated with a culture that was contaminated with Candida parapsilosis”
- Peterson 2009: Notes AEs “linked to recognized effects of HRV infections (for example, earache, pharyngitis and headache)” but does not provide detail on them

#

# PRISMA Checklists

## PRISMA Checklist

| **Section and Topic** | **Item #** | **Checklist item** | **Location where item is reported** |
| --- | --- | --- | --- |
| **TITLE** | | |  |
| Title | 1 | Identify the report as a systematic review. | Title |
| **ABSTRACT** | | |  |
| Abstract | 2 | See the PRISMA 2020 for Abstracts checklist. | Abstract |
| **INTRODUCTION** | | |  |
| Rationale | 3 | Describe the rationale for the review in the context of existing knowledge. | Introduction |
| Objectives | 4 | Provide an explicit statement of the objective(s) or question(s) the review addresses. | Introduction |
| **METHODS** | | |  |
| Eligibility criteria | 5 | Specify the inclusion and exclusion criteria for the review and how studies were grouped for the syntheses. | Sec 2.2, 2.3 |
| Information sources | 6 | Specify all databases, registers, websites, organisations, reference lists and other sources searched or consulted to identify studies. Specify the date when each source was last searched or consulted. | Sec 2.1, Table 1 |
| Search strategy | 7 | Present the full search strategies for all databases, registers and websites, including any filters and limits used. | Sec 2.1, Table 1 |
| Selection process | 8 | Specify the methods used to decide whether a study met the inclusion criteria of the review, including how many reviewers screened each record and each report retrieved, whether they worked independently, and if applicable, details of automation tools used in the process. | Sec 2.2, 2.3 |
| Data collection process | 9 | Specify the methods used to collect data from reports, including how many reviewers collected data from each report, whether they worked independently, any processes for obtaining or confirming data from study investigators, and if applicable, details of automation tools used in the process. | Sec 2.4 |
| Data items | 10a | List and define all outcomes for which data were sought. Specify whether all results that were compatible with each outcome domain in each study were sought (e.g. for all measures, time points, analyses), and if not, the methods used to decide which results to collect. | Sec 2.5 |
|  | 10b | List and define all other variables for which data were sought (e.g. participant and intervention characteristics, funding sources). Describe any assumptions made about any missing or unclear information. | Sec 2.5, Supplementary Materials (Methods) |
| Study risk of bias assessment | 11 | Specify the methods used to assess risk of bias in the included studies, including details of the tool(s) used, how many reviewers assessed each study and whether they worked independently, and if applicable, details of automation tools used in the process. | Supplementary Materials (Methods) |
| Effect measures | 12 | Specify for each outcome the effect measure(s) (e.g. risk ratio, mean difference) used in the synthesis or presentation of results. | N/A |
| Synthesis methods | 13a | Describe the processes used to decide which studies were eligible for each synthesis (e.g. tabulating the study intervention characteristics and comparing against the planned groups for each synthesis (item #5)). | N/A |
|  | 13b | Describe any methods required to prepare the data for presentation or synthesis, such as handling of missing summary statistics, or data conversions. | N/A |
|  | 13c | Describe any methods used to tabulate or visually display results of individual studies and syntheses. | N/A |
|  | 13d | Describe any methods used to synthesize results and provide a rationale for the choice(s). If meta-analysis was performed, describe the model(s), method(s) to identify the presence and extent of statistical heterogeneity, and software package(s) used. | N/A |
|  | 13e | Describe any methods used to explore possible causes of heterogeneity among study results (e.g. subgroup analysis, meta-regression). | N/A |
|  | 13f | Describe any sensitivity analyses conducted to assess robustness of the synthesized results. | N/A |
| Reporting bias assessment | 14 | Describe any methods used to assess risk of bias due to missing results in a synthesis (arising from reporting biases). | N/A |
| Certainty assessment | 15 | Describe any methods used to assess certainty (or confidence) in the body of evidence for an outcome. | N/A |
| **RESULTS** | | |  |
| Study selection | 16a | Describe the results of the search and selection process, from the number of records identified in the search to the number of studies included in the review, ideally using a flow diagram. | Sec 3.1, Figure 1 |
|  | 16b | Cite studies that might appear to meet the inclusion criteria, but which were excluded, and explain why they were excluded. | Supplementary Materials (Excluded Studies) |
| Study characteristics | 17 | Cite each included study and present its characteristics. | Supplementary Materials (Included Studies), Dataset |
| Risk of bias in studies | 18 | Present assessments of risk of bias for each included study. | N/A |
| Results of individual studies | 19 | For all outcomes, present, for each study: (a) summary statistics for each group (where appropriate) and (b) an effect estimate and its precision (e.g. confidence/credible interval), ideally using structured tables or plots. | Sec 3.2-6 |
| Results of syntheses | 20a | For each synthesis, briefly summarise the characteristics and risk of bias among contributing studies. | N/A |
|  | 20b | Present results of all statistical syntheses conducted. If meta-analysis was done, present for each the summary estimate and its precision (e.g. confidence/credible interval) and measures of statistical heterogeneity. If comparing groups, describe the direction of the effect. | N/A |
|  | 20c | Present results of all investigations of possible causes of heterogeneity among study results. | N/A |
|  | 20d | Present results of all sensitivity analyses conducted to assess the robustness of the synthesized results. | N/A |
| Reporting biases | 21 | Present assessments of risk of bias due to missing results (arising from reporting biases) for each synthesis assessed. | N/A |
| Certainty of evidence | 22 | Present assessments of certainty (or confidence) in the body of evidence for each outcome assessed. | N/A |
| **DISCUSSION** | | |  |
| Discussion | 23a | Provide a general interpretation of the results in the context of other evidence. | Sec 4 |
|  | 23b | Discuss any limitations of the evidence included in the review. | Sec 4 |
|  | 23c | Discuss any limitations of the review processes used. | Sec 4 |
|  | 23d | Discuss implications of the results for practice, policy, and future research. | Sec 4, 5 |
| **OTHER INFORMATION** | | |  |
| Registration and protocol | 24a | Provide registration information for the review, including register name and registration number, or state that the review was not registered. | Sec 2.1 |
|  | 24b | Indicate where the review protocol can be accessed, or state that a protocol was not prepared. | Review Protocol |
|  | 24c | Describe and explain any amendments to information provided at registration or in the protocol. | Amended Preregistration |
| Support | 25 | Describe sources of financial or non-financial support for the review, and the role of the funders or sponsors in the review. | Conflict of Interest Disclosures |
| Competing interests | 26 | Declare any competing interests of review authors. | Competing Interests |
| Availability of data, code and other materials | 27 | Report which of the following are publicly available and where they can be found: template data collection forms; data extracted from included studies; data used for all analyses; analytic code; any other materials used in the review. | Dataset |

*From:*  Page MJ, McKenzie JE, Bossuyt PM, Boutron I, Hoffmann TC, Mulrow CD, et al. The PRISMA 2020 statement: an updated guideline for reporting systematic reviews. BMJ 2021;372:n71. doi: 10.1136/bmj.n71

## PRISMA 2020 for Abstracts Checklist

| **Section and Topic** | **Item #** | **Checklist item** | **Reported (Yes/No)** |
| --- | --- | --- | --- |
| **TITLE** | | |  |
| Title | 1 | Identify the report as a systematic review. | Yes |
| **BACKGROUND** | | |  |
| Objectives | 2 | Provide an explicit statement of the main objective(s) or question(s) the review addresses. | Yes |
| **METHODS** | | |  |
| Eligibility criteria | 3 | Specify the inclusion and exclusion criteria for the review. | Yes |
| Information sources | 4 | Specify the information sources (e.g. databases, registers) used to identify studies and the date when each was last searched. | Yes |
| Risk of bias | 5 | Specify the methods used to assess risk of bias in the included studies. | N/A |
| Synthesis of results | 6 | Specify the methods used to present and synthesise results. | N/A |
| **RESULTS** | | |  |
| Included studies | 7 | Give the total number of included studies and participants and summarise relevant characteristics of studies. | Yes |
| Synthesis of results | 8 | Present results for main outcomes, preferably indicating the number of included studies and participants for each. If meta-analysis was done, report the summary estimate and confidence/credible interval. If comparing groups, indicate the direction of the effect (i.e. which group is favoured). | Yes |
| **DISCUSSION** | | |  |
| Limitations of evidence | 9 | Provide a brief summary of the limitations of the evidence included in the review (e.g. study risk of bias, inconsistency and imprecision). | No |
| Interpretation | 10 | Provide a general interpretation of the results and important implications. | Yes |
| **OTHER** | | |  |
| Funding | 11 | Specify the primary source of funding for the review. | Yes |
| Registration | 12 | Provide the register name and registration number. | Yes |

*From:*  Page MJ, McKenzie JE, Bossuyt PM, Boutron I, Hoffmann TC, Mulrow CD, et al. The PRISMA 2020 statement: an updated guideline for reporting systematic reviews. BMJ 2021;372:n71. doi: 10.1136/bmj.n71

# Supplementary Tables

## Data Categorization

Column AV in the “Included” tab of the dataset contains category tags that were applied to each study to allow for automatic table generation based on different criteria. The following category tags were used:

- Challenge: describes a traditional challenge trial
- Prechallenge: describes an immunization phase or a vaccination phase that occurred prior to a traditional challenge phase
- Rechallenge: describes a cohort of volunteers that have been previously exposed to a challenge agent (in a previous study or in an earlier challenge phase of the current study), that were then challenged again (either with the same strain or a different strain of the challenge agent)
- Attenuated: describes a challenge with an attenuated challenge agent
- Vaccine: describes a challenge with a vaccine
- Summed: previously extracted data from multiple phases, studies, or cohorts that was summed to be treated as a single study
- NAE: studies where AEs were not mentioned/defined, and other reported data (symptoms, illness, etc.) was extracted instead
- NSAE: studies where SAEs were not mentioned in the text, and the current FDA definition of SAEs was manually applied to reported symptom/illness data in order to identify possible SAEs
- Excluded: studies that were excluded for any reason

## Supplementary Table 1 - Reasons for Exclusion

| **Reasons for exclusion** | **Excluded during screening, n** | **Excluded during full text review, n** |
| --- | --- | --- |
| Not challenge trials | 1053 | 28 |
| Review, guidelines, editorials, or comments | 974 | 2 |
| Animal studies | 327 | 0 |
| Not in English | 38 | 1 |
| Case report | 21 | 1 |
| Duplicate | 1 | 47 |
| Pre-1980s | 0 | 30 |
| Uses previously published data | 0 | 26 |
| Unpublished data | 0 | 6 |
| Unable to locate or access (full text not available) | 0 | 5 |
| Insufficient data | 0 | 1 |
| Describes protocol for a future study | 0 | 1 |
| Total | 2414 | 148 |

## Supplementary Table 2 - Reasons for Exclusion during Full-Text Review (By Method of Identification)

| **Reasons for exclusion** | **Excluded (search results), n** | **Excluded (other reports), n** |
| --- | --- | --- |
| Not challenge trials | 26 | 0 |
| Duplicates | 19 | 28 |
| Uses previously published data | 18 | 8 |
| Pre-1980s | 14 | 16 |
| Unpublished data | 1 | 5 |
| Unable to locate/access (no full text available) | 1 | 4 |
| Describes protocol for future study | 1 | 0 |
| Reviews | 0 | 2 |
| Not challenge trials | 0 | 2 |
| Case reports | 0 | 1 |
| Insufficient data | 0 | 1 |
| Not in English | 0 | 1 |
| Total | 80 | 68 |

## Supplementary Table 3 - Number of Studies, Number of Participants, Number of Infections, Data Reporting, and Database Registration in Published HCTs by Decade (Rechallenges) (Excluded Tags: Challenge, Prechallenge, Excluded)

| **Decade** | **Studies, n** | **Participants challenged, n** | **Challenged participants diagnosed with infection, n** | **Studies that do not define AEs, n (%)** | **Studies with unclear AE data, n (%)** | **Studies with no AE data, n (%)** | **Studies that do not mention SAEs, n (%)** | **Studies with no SAE data, n (%)** | **Studies registered in a database, n (%)** |
| --- | --- | --- | --- | --- | --- | --- | --- | --- | --- |
| **1980s** | 2 | 28 | 15 | 2 (100.0) | 0 (0.0) | 0 (0.0) | 2 (100.0) | 0 (0.0) | 0 (0.0) |
| **1990s** | 9 | 69 | 54-55^a^ | 8 (88.9) | 0 (0.0) | 8 (88.9) | 1 (11.1) | 8 (88.9) | 0 (0.0) |
| **2000s** | 3 | 32 | 20 | 3 (100.0) | 0 (0.0) | 3 (100.0) | 0 (0.0) | 3 (100.0) | 0 (0.0) |
| **2010s** | 8 | 203 | 89 | 1 (12.5) | 1 (12.5) | 4 (50.0) | 1 (12.5) | 6 (75.0) | 6 (75.0) |
| **2020s** | 2 | 86 | 40 | 1 (50.0) | 2 (100.) | 0 (0.0) | 0 (0.0) | 0 (0.0) | 2 (100.0) |
| **Total** | 24 | 418 | 218-219^a^ | 15 (62.5) | 3 (12.5) | 15 (62.5) | 4 (16.7) | 17 (70.8) | 8 (33.3) |

^a^A range of values is given to account for unclear data reporting by one study.

## Supplementary Table 4 - Adverse Events in Published HCTs by Decade (Rechallenges) (Excluded Tags: Challenge, Prechallenge, Excluded)

| **Decade** | **Studies^a^, n** | **Participants challenged, n** | **Challenged participants with AEs (minimum^b^), n (%)** | **Challenged participants with AEs (maximum^b^), n (%)** |
| --- | --- | --- | --- | --- |
| **1980s** | 2 | 28 | 2 (7.1) | 2 (7.1) |
| **1990s** | 1 | 13 | 0 (0.0) | 0 (0.0) |
| **2000s** | 0 | 0 | 0 (0.0) | 0 (0.0) |
| **2010s** | 4 | 35 | 17 (48.6) | 20 (57.1) |
| **2020s** | 2 | 86 | 8 (9.3) | 86 (100.0) |
| **Total** | 9 | 162 | 27 (16.7) | 108 (66.7) |

^a^15 studies that did not report AE data are excluded.

^b^Minimum and maximum values are given to account for unclear data reporting by some studies.

## Supplementary Table 5 - Severe Adverse Events in Published HCTs by Decade (Rechallenges) (Excluded Tags: Challenge, Prechallenge, Excluded)

| **Decade** | **Studies^a^, n** | **Participants challenged, n** | **Challenged participants with severe or very severe (≥grade 3) AEs (minimum^b^), n (%)** | **Challenged participants with severe or very severe (≥grade 3) AEs (maximum^b^), n (%)** |
| --- | --- | --- | --- | --- |
| **1980s** | 0 | 0 | 0 (0.0) | 0 (0.0) |
| **1990s** | 0 | 0 | 0 (0.0) | 0 (0.0) |
| **2000s** | 0 | 0 | 0 (0.0) | 0 (0.0) |
| **2010s** | 3 | 31 | 5 (16.1) | 14 (45.2) |
| **2020s** | 0 | 0 | 0 (0.0) | 0 (0.0) |
| **Total** | 3 | 31 | 5 (16.1) | 14 (45.2) |

^a^21 studies that did not report severe AE data are excluded.

^b^Minimum and maximum values are given to account for unclear data reporting by some studies.

## Supplementary Table 6 - Serious Adverse Events in Published HCTs by Decade (Rechallenges) (Excluded Tags: Challenge, Prechallenge, Excluded)

| **Decade** | **Studies^a^, n** | **Participants challenged, n** | **Challenged participants with SAEs, n (%)** |
| --- | --- | --- | --- |
| **1980s** | 2 | 28 | 0 (0.0) |
| **1990s** | 1 | 13 | 0 (0.0) |
| **2000s** | 0 | 0 | 0 (0.0) |
| **2010s** | 2 | 19 | 0 (0.0) |
| **2020s** | 2 | 86 | 1 (1.2) |
| **Total** | 7 | 146 | 1 (0.7) |

^a^17 studies that did not report SAE data are excluded.

## Supplementary Table 7 - Descriptions of Additional Serious Adverse Events (Not Related to Challenge) in Published HCTs by Pathogen Category

| **Pathogen category** | **Participants with ≥1 SAE, n** | **Description** | **Outcomes** | **Dataset file name** |
| --- | --- | --- | --- | --- |
| **Escherichia coli** |  |  |  |  |
|  | 1* | "There were no serious AEs following vaccination, but one occurred in a placebo recipient during long-term follow-up postchallenge. This event was not considered to be related to participation in the study." | ND | Darsley 2012 |
| **Neisseria lactamica** |  |  |  |  |
|  | 4* | "Four participants experienced serious adverse events but none were related to the study." | ND | Deasy 2015 |
| **Norovirus** |  |  |  |  |
|  | 1* | "There was 1 serious adverse event during the study and it was assessed as unrelated. A placebo recipient developed a partial retinal detachment 74 days after receipt of study product." | ND | Atmar 2014 |
| **Plasmodium spp.** |  |  |  |  |
|  | 1* | "All volunteers successfully recovered clinically within 2–3 days without any serious AE. None of the volunteers required hospitalization for malaria. A volunteer presented with severe diarrhea and abdominal pain (Grade 4) on day 18 and was diagnosed with parasitic gastroenteritis (hookworms and trichuriasis) and was held under observation for <24 hours in the hospital, requiring intravenous rehydration and pain management." | ND | Arévalo-Herrera 2014 |
|  | 1* | "One SAE (ductal carcinoma in situ of the breast) occurred during the study and it was determined that the event was not related to the study vaccine or CHMI." | ND | Bennett 2016 |
|  | 1* | "The only serious adverse event (recurrent tonsillitis) was considered unrelated to the clinical trial treatment." | ND | McCarthy 2016a |
|  | 1* | "One subject was diagnosed with colon cancer and did not participate in CHMI." | ND | Seder 2013 |
|  | 1* | "One adverse event (renal colic) was classified as serious; however, this event was determined not to be related to study treatments." | "All adverse events had resolved by the end of study." | Smith 2017 |
|  | 1 | "During follow-up, one serious adverse event occurred in cohort 1A: a bilateral pulmonary embolism occurred in a non-smoking woman under oral contraceptive treatment 34 days after DSM265 administration while she was on a transatlantic flight." | "The volunteer never became parasitaemic and recovered fully, except for a grade 1 sinus tachycardia, which still persisted at the last follow-up visit." | Sulyok 2017 |
| **Rhinovirus** |  |  |  |  |
|  | 1* | "One serious adverse event (SAE) was reported in the 1 TCID50 group. The SAE was a Grade 4 self-limiting raised creatine phosphokinase considered to be of medical importance, but not considered to be related to the Challenge Virus, study procedures, concomitant medications or study assessments." | "No action was taken and the SAE resolved." | Fullen 2016b |
| **Salmonella spp.** |  |  |  |  |
|  | 1* | "One serious adverse event was recorded (appendicitis 6 months after S. Paratyphi A challenge) and was assessed as being unrelated to study procedures." | ND | Dobinson 2017 |
|  | 1 | "Generalised tonic clonic seizure": "Occurred 9 months after completion of challenge. Reported a history of childhood seizures that was not disclosed at screening or on available medical records. Commenced on anticonvulsants and ongoing follow up with neurology at end of study" | ND | Gibani 2019 |
|  | 1* | "Deliberate self harm": "Episode of deliberate self-harm 6 months after completion of challenge, attributed to an acute life stressor. No specific treatment was administered." | ND | Gibani 2019 |
|  | 1* | "Episode of collapse with loss of consciousness 12 months after challenge. Reported left arm and left leg weakness 24 hours’ duration. Diagnosed as possible generalised seizure following neurology review. Instructed not to drive for 6/12." | "No medication prescribed, and no subsequent events." | Gibani 2020 |
|  | 1* | "Right loin pain with evidence of renal calculus on CT KUB. Diagnosed after enrolment but prior to challenge. Withdrawn from study and not challenged." | ND | Gibani 2020 |
|  | 1* | "One Vi-TT participant was diagnosed with inflammatory bowel disease and withdrawn from the study (symptoms preceded study enrolment)" | ND | Jin 2017 |
|  | 2* | "two Vi-PS participants were hospitalised (urinary retention and semi-elective tonsillectomy)" | ND | Jin 2017 |
| **Shigella spp.** |  |  |  |  |
|  | 1* | "In the Placebo group, one participant experienced four SAEs following the challenge dose: deep vein thrombosis (moderate intensity) and pelvic venous thrombosis (severe) at day 107, haematoma (severe) at day 121, and carotid artery aneurysm at day 130 from challenge." | "All events were assessed by the investigator as unrelated to any study treatment and were resolved." | Frenck 2021 |

*Considered unrelated to study participation.

## Supplementary Table 8 - Number of Published HCTS, Number of Participants, Number Infected, and Number with Serious Adverse Events by Pathogen Category (Rechallenges) (Excluded Tags: Challenge, Prechallenge, Excluded)

| **Pathogen category** | **Studies, n** | **Participants challenged across all studies, n** | **Challenged participants diagnosed with infection across all studies, n (%)** | **Studies that reported SAEs, n** | **Participants challenged across studies that reported SAEs, n** | **Challenged participants with SAEs across studies that reported SAEs, n (%)** |
| --- | --- | --- | --- | --- | --- | --- |
| **Campylobacter jejuni** | 2 | 24 | 18 (75.0) | 2 | 24 | 0 (0.0) |
| **Escherichia coli** | 4 | 40 | 33 (82.5) | 1 | 11 | 0 (0.0) |
| **Haemophilus ducreyi** | 5 | 54 | 27 (50.0) | 0 | 0 | 0 (0.0) |
| **Influenza viruses** | 2 | 32 | 16 (50.0) | 2 | 32 | 0 (0.0) |
| **Necator americanus** | 2 | 17 | 14 (82.4) | 0 | 0 | 0 (0.0) |
| **Neisseria lactamica** | 1 | 127 | 44 (34.6) | 0 | 0 | 0 (0.0) |
| **Plasmodium spp.** | 6 | 43 | 13 (30.2) | 1 | 4 | 0 (0.0) |
| **Salmonella spp.** | 1 | 75 | 35 (46.7) | 1 | 75 | 1 (1.3) |
| **Shigella spp.** | 1 | 6 | 1 (16.7) | 0 | 0 | 0 (0.0) |
| **Total** | 24 | 418 | 201 (48.1) | 7 | 146 | 1 (0.7) |

## Supplementary Table 9 - Adverse Events in Published HCTs by Decade (Excluding manual AE counts) (Excluded Tags: Prechallenge, Rechallenge, NAE, Excluded)

| **Decade** | **Studies^a^, n** | **Participants challenged, n** | **Challenged participants with AEs (minimum^b^), n (%)** | **Challenged participants with AEs (maximum^b^), n (%)** |
| --- | --- | --- | --- | --- |
| **1980s** | 0 | 0 | 0 (0.0) | 0 (0.0) |
| **1990s** | 3 | 518 | 136 (26.3) | 153 (29.5) |
| **2000s** | 16 | 1627 | 524 (32.2) | 768 (47.2) |
| **2010s** | 71 | 2697 | 1324 (49.1) | 1939 (71.9) |
| **2020s** | 20 | 879 | 417 (47.4) | 707 (80.4) |
| **Total** | 110 | 5721 | 2401 (42.0) | 3567 (62.3) |

^a^93 studies that did not report AE data are excluded.

^b^Minimum and maximum values are given to account for unclear data reporting by some studies.

## Supplementary Table 10 - Serious Adverse Events in Published HCTs by Decade (Excluding manual SAE counts) (Excluded Tags: Prechallenge, Rechallenge, NSAE, Excluded)

| **Decade** | **Studies^a^, n** | **Challenged participants, n** | **Challenged participants with SAEs, n (%)** |
| --- | --- | --- | --- |
| **1980s** | 2 | 17 | 0 (0.0) |
| **1990s** | 1 | 15 | 0 (0.0) |
| **2000s** | 14 | 796 | 1 (0.1) |
| **2010s** | 65 | 2832 | 9 (0.3) |
| **2020s** | 21 | 931 | 2 (0.2) |
| **Total** | 103 | 4591 | 12 (0.3) |

^a^97 studies that did not report SAE data are excluded.

# Supplementary References

## Reference List of Included Studies

The following is a list of all articles describing included studies. Characteristics of each study are provided in the online dataset.

1. Abisheganaden JA, Avila PC, Kishiyama JL, et al. Effect of clarithromycin on experimental rhinovirus-16 colds: a randomized, double-blind, controlled trial. Am J Med **2000**; 108:453–459.

2. Achan J, Reuling IJ, Yap XZ, et al. Serologic Markers of Previous Malaria Exposure and Functional Antibodies Inhibiting Parasite Growth Are Associated With Parasite Kinetics Following a Plasmodium falciparum Controlled Human Infection. Clin Infect Dis **2020**; 70:2544–2552.

3. Adler H, German EL, Mitsi E, et al. Experimental Human Pneumococcal Colonization in Older Adults Is Feasible and Safe, Not Immunogenic. Am J Respir Crit Care Med **2021**; 203:604–613.

4. Al-Nakib W, Higgins PG, Barrow I, Tyrrell DA, Lenox-Smith I, Ishitsuka H. Intranasal chalcone, Ro 09-0410, as prophylaxis against rhinovirus infection in human volunteers. J Antimicrob Chemother **1987**; 20:887–892.

5. Al-Nakib W, Higgins PG, Willman J, et al. Prevention and treatment of experimental influenza A virus infection in volunteers with a new antiviral ICI 130,685. J Antimicrob Chemother **1986**; 18:119–129.

6. Al-Tawfiq JA, Bauer ME, Fortney KR, et al. A pilus-deficient mutant of Haemophilus ducreyi is virulent in the human model of experimental infection. J Infect Dis **2000**; 181:1176–1179.

7. Al-Tawfiq JA, Fortney KR, Katz BP, Hood AF, Elkins C, Spinola SM. An isogenic hemoglobin receptor-deficient mutant of Haemophilus ducreyi is attenuated in the human model of experimental infection. J Infect Dis **2000**; 181:1049–1054.

8. Al-Tawfiq JA, Palmer KL, Chen CY, et al. Experimental infection of human volunteers with Haemophilus ducreyi does not confer protection against subsequent challenge. J Infect Dis **1999**; 179:1283–1287.

9. Al-Tawfiq JA, Thornton AC, Katz BP, et al. Standardization of the experimental model of Haemophilus ducreyi infection in human subjects. J Infect Dis **1998**; 178:1684–1687.

10. Alper CM, Doyle WJ, Skoner DP, et al. Prechallenge antibodies: moderators of infection rate, signs, and symptoms in adults experimentally challenged with rhinovirus type 39. Laryngoscope **1996**; 106:1298–1305.

11. Anderson MJ, Higgins PG, Davis LR, et al. Experimental parvoviral infection in humans. J Infect Dis **1985**; 152:257–265.

12. Arévalo-Herrera M, Forero-Peña DA, Rubiano K, et al. Plasmodium vivax sporozoite challenge in malaria-naïve and semi-immune Colombian volunteers. PLoS One **2014**; 9:e99754.

13. Arévalo-Herrera M, Vásquez-Jiménez JM, Lopez-Perez M, et al. Protective Efficacy of Plasmodium vivax Radiation-Attenuated Sporozoites in Colombian Volunteers: A Randomized Controlled Trial. PLoS Negl Trop Dis **2016**; 10:e0005070.

14. Atmar RL, Opekun AR, Gilger MA, et al. Norwalk virus shedding after experimental human infection. Emerg Infect Dis **2008**; 14:1553–1557.

15. Atmar RL, Opekun AR, Gilger MA, et al. Determination of the 50% human infectious dose for Norwalk virus. J Infect Dis **2014**; 209:1016–1022.

16. Ballou WR, Hoffman SL, Sherwood JA, et al. Safety and efficacy of a recombinant DNA Plasmodium falciparum sporozoite vaccine. Lancet **1987**; 1:1277–1281.

17. Banks KE, Fortney KR, Baker B, et al. The enterobacterial common antigen-like gene cluster of Haemophilus ducreyi contributes to virulence in humans. J Infect Dis **2008**; 197:1531–1536.

18. Barroso L, Treanor J, Gubareva L, Hayden FG. Efficacy and tolerability of the oral neuraminidase inhibitor peramivir in experimental human influenza: randomized, controlled trials for prophylaxis and treatment. Antivir Ther **2005**; 10:901–910.

19. Bastiaens GJH, van Meer MPA, Scholzen A, et al. Safety, Immunogenicity, and Protective Efficacy of Intradermal Immunization with Aseptic, Purified, Cryopreserved Plasmodium falciparum Sporozoites in Volunteers Under Chloroquine Prophylaxis: A Randomized Controlled Trial. Am J Trop Med Hyg **2016**; 94:663–673.

20. Bauer ME, Townsend CA, Doster RS, et al. A fibrinogen-binding lipoprotein contributes to the virulence of Haemophilus ducreyi in humans. J Infect Dis **2009**; 199:684–692.

21. Bennett JW, Yadava A, Tosh D, et al. Phase 1/2a Trial of Plasmodium vivax Malaria Vaccine Candidate VMP001/AS01B in Malaria-Naive Adults: Safety, Immunogenicity, and Efficacy. PLoS Negl Trop Dis **2016**; 10:e0004423.

22. Bernstein DI, Atmar RL, Lyon GM, et al. Norovirus vaccine against experimental human GII.4 virus illness: a challenge study in healthy adults. J Infect Dis **2015**; 211:870–878.

23. Bijker EM, Bastiaens GJH, Teirlinck AC, et al. Protection against malaria after immunization by chloroquine prophylaxis and sporozoites is mediated by preerythrocytic immunity. Proc Natl Acad Sci U S A **2013**; 110:7862–7867.

24. Bijker EM, Schats R, Obiero JM, et al. Sporozoite immunization of human volunteers under mefloquine prophylaxis is safe, immunogenic and protective: a double-blind randomized controlled clinical trial. PLoS One **2014**; 9:e112910.

25. Bijker EM, Teirlinck AC, Schats R, et al. Cytotoxic markers associate with protection against malaria in human volunteers immunized with Plasmodium falciparum sporozoites. J Infect Dis **2014**; 210:1605–1615.

26. Bjornson AB, Mellencamp MA, Schiff GM. Complement is activated in the upper respiratory tract during influenza virus infection. Am Rev Respir Dis **1991**; 143:1062–1066.

27. Black RE, Levine MM, Clements ML, Cisneros L, Daya V. Treatment of experimentally induced enterotoxigenic Escherichia coli diarrhea with trimethoprim, trimethoprim-sulfamethoxazole, or placebo. Rev Infect Dis **1982**; 4:540–545.

28. Black RE, Levine MM, Clements ML, et al. Prevention of shigellosis by a Salmonella typhi-Shigella sonnei bivalent vaccine. J Infect Dis **1987**; 155:1260–1265.

29. Black RE, Levine MM, Clements ML, Hughes TP, Blaser MJ. Experimental Campylobacter jejuni infection in humans. J Infect Dis **1988**; 157:472–479.

30. Bodhidatta L, Pitisuttithum P, Chamnanchanant S, et al. Establishment of a Shigella sonnei human challenge model in Thailand. Vaccine **2012**; 30:7040–7045.

31. Bong CT, Throm RE, Fortney KR, et al. DsrA-deficient mutant of Haemophilus ducreyi is impaired in its ability to infect human volunteers. Infect Immun **2001**; 69:1488–1491.

32. Bong CTH, Fortney KR, Katz BP, et al. A superoxide dismutase C mutant of Haemophilus ducreyi is virulent in human volunteers. Infect Immun **2002**; 70:1367–1371.

33. Buchman CA, Doyle WJ, Pilcher O, Gentile DA, Skoner DP. Nasal and otologic effects of experimental respiratory syncytial virus infection in adults. Am J Otolaryngol **2002**; 23:70–75.

34. Calfee DP, Peng AW, Cass LM, Lobo M, Hayden FG. Safety and efficacy of intravenous zanamivir in preventing experimental human influenza A virus infection. Antimicrob Agents Chemother **1999**; 43:1616–1620.

35. Calhoun WJ, Swenson CA, Dick EC, Schwartz LB, Lemanske RF, Busse WW. Experimental rhinovirus 16 infection potentiates histamine release after antigen bronchoprovocation in allergic subjects. Am Rev Respir Dis **1991**; 144:1267–1273.

36. Cannon JG, Tompkins RG, Gelfand JA, et al. Circulating interleukin-1 and tumor necrosis factor in septic shock and experimental endotoxin fever. J Infect Dis **1990**; 161:79–84.

37. Cate TR, Couch RB. Live influenza A/Victoria/75 (H3N2) virus vaccines: reactogenicity, immunogenicity, and protection against wild-type virus challenge. Infect Immun **1982**; 38:141–146.

38. Chakraborty S, Harro C, DeNearing B, et al. Impact of lower challenge doses of enterotoxigenic Escherichia coli on clinical outcome, intestinal colonization and immune responses in adult volunteers. PLoS Negl Trop Dis **2018**; 12:e0006442.

39. Chapman PR, Webster R, Giacomin P, et al. Vaccination of human participants with attenuated Necator americanus hookworm larvae and human challenge in Australia: a dose-finding study and randomised, placebo-controlled, phase 1 trial. Lancet Infect Dis **2021**; 21:1725–1736.

40. Chappell CL, Okhuysen PC, Langer-Curry R, et al. Cryptosporidium hominis: experimental challenge of healthy adults. Am J Trop Med Hyg **2006**; 75:851–857.

41. Chen WH, Cohen MB, Kirkpatrick BD, et al. Single-dose Live Oral Cholera Vaccine CVD 103-HgR Protects Against Human Experimental Infection With Vibrio cholerae O1 El Tor. Clin Infect Dis **2016**; 62:1329–1335.

42. Chuang I, Sedegah M, Cicatelli S, et al. DNA prime/Adenovirus boost malaria vaccine encoding P. falciparum CSP and AMA1 induces sterile protection associated with cell-mediated immunity. PLoS One **2013**; 8:e55571.

43. Chulay JD, Schneider I, Cosgriff TM, et al. Malaria transmitted to humans by mosquitoes infected from cultured Plasmodium falciparum. Am J Trop Med Hyg **1986**; 35:66–68.

44. Clements ML, Belshe RB, King J, et al. Evaluation of bovine, cold-adapted human, and wild-type human parainfluenza type 3 viruses in adult volunteers and in chimpanzees. J Clin Microbiol **1991**; 29:1175–1182.

45. Clements ML, Betts RF, Murphy BR. Advantage of live attenuated cold-adapted influenza A virus over inactivated vaccine for A/Washington/80 (H3N2) wild-type virus infection. Lancet **1984**; 1:705–708.

46. Clements ML, Betts RF, Tierney EL, Murphy BR. Resistance of adults to challenge with influenza A wild-type virus after receiving live or inactivated virus vaccine. J Clin Microbiol **1986**; 23:73–76.

47. Clements ML, O’Donnell S, Levine MM, Chanock RM, Murphy BR. Dose response of A/Alaska/6/77 (H3N2) cold-adapted reassortant vaccine virus in adult volunteers: role of local antibody in resistance to infection with vaccine virus. Infect Immun **1983**; 40:1044–1051.

48. Clements ML, Snyder MH, Sears SD, Maassab HF, Murphy BR. Evaluation of the infectivity, immunogenicity, and efficacy of live cold-adapted influenza B/Ann Arbor/1/86 reassortant virus vaccine in adult volunteers. J Infect Dis **1990**; 161:869–877.

49. Clements ML, Wisseman CL, Woodward TE, et al. Reactogenicity, immunogenicity, and efficacy of a chick embryo cell-derived vaccine for Rocky Mountain spotted fever. J Infect Dis **1983**; 148:922–930.

50. Cohen S, Doyle WJ, Skoner DP, Rabin BS, Gwaltney JM. Social ties and susceptibility to the common cold. JAMA **1997**; 277:1940–1944.

51. Cohen S, Doyle WJ, Skoner DP. Psychological stress, cytokine production, and severity of upper respiratory illness. Psychosom Med **1999**; 61:175–180.

52. Collins AM, Wright AD, Mitsi E, et al. First human challenge testing of a pneumococcal vaccine. Double-blind randomized controlled trial. Am J Respir Crit Care Med **2015**; 192:853–858.

53. Collins KA, Wang CY, Adams M, et al. A controlled human malaria infection model enabling evaluation of transmission-blocking interventions. J Clin Invest **2018**; 128:1551–1562.

54. Coster TS, Hoge CW, VanDeVerg LL, et al. Vaccination against shigellosis with attenuated Shigella flexneri 2a strain SC602. Infect Immun **1999**; 67:3437–3443.

55. Coster TS, Wolf MK, Hall ER, et al. Immune response, ciprofloxacin activity, and gender differences after human experimental challenge by two strains of enterotoxigenic Escherichia coli. Infect Immun **2007**; 75:252–259.

56. Cremers AJ, Zomer AL, Gritzfeld JF, et al. The adult nasopharyngeal microbiome as a determinant of pneumococcal acquisition. Microbiome **2014**; 2:44.

57. Croese J, O’neil J, Masson J, et al. A proof of concept study establishing Necator americanus in Crohn’s patients and reservoir donors. Gut **2006**; 55:136–137.

58. Croese J, Giacomin P, Navarro S, et al. Experimental hookworm infection and gluten microchallenge promote tolerance in celiac disease. J Allergy Clin Immunol **2015**; 135:508–516.

59. Cummings JF, Spring MD, Schwenk RJ, et al. Recombinant Liver Stage Antigen-1 (LSA-1) formulated with AS01 or AS02 is safe, elicits high titer antibody and induces IFN-gamma/IL-2 CD4+ T cells but does not protect against experimental Plasmodium falciparum infection. Vaccine **2010**; 28:5135–5144.

60. Darsley MJ, Chakraborty S, DeNearing B, et al. The oral, live attenuated enterotoxigenic Escherichia coli vaccine ACE527 reduces the incidence and severity of diarrhea in a human challenge model of diarrheal disease. Clin Vaccine Immunol **2012**; 19:1921–1931.

61. Darton TC, Jones C, Blohmke CJ, et al. Using a Human Challenge Model of Infection to Measure Vaccine Efficacy: A Randomised, Controlled Trial Comparing the Typhoid Vaccines M01ZH09 with Placebo and Ty21a. PLoS Negl Trop Dis **2016**; 10:e0004926.

62. Davis JR, Cortese JF, Herrington DA, et al. Plasmodium falciparum: in vitro characterization and human infectivity of a cloned line. Exp Parasitol **1992**; 74:159–168.

63. de Graaf H, Ibrahim M, Hill AR, et al. Controlled Human Infection With Bordetella pertussis Induces Asymptomatic, Immunizing Colonization. Clin Infect Dis **2020**; 71:403–411.

64. Deasy AM, Guccione E, Dale AP, et al. Nasal Inoculation of the Commensal Neisseria lactamica Inhibits Carriage of Neisseria meningitidis by Young Adults: A Controlled Human Infection Study. Clin Infect Dis **2015**; 60:1512–1520.

65. Dejon-Agobe JC, Ateba-Ngoa U, Lalremruata A, et al. Controlled Human Malaria Infection of Healthy Adults With Lifelong Malaria Exposure to Assess Safety, Immunogenicity, and Efficacy of the Asexual Blood Stage Malaria Vaccine Candidate GMZ2. Clin Infect Dis **2019**; 69:1377–1384.

66. DeVincenzo JP, McClure MW, Symons JA, et al. Activity of Oral ALS-008176 in a Respiratory Syncytial Virus Challenge Study. N Engl J Med **2015**; 373:2048–2058.

67. DeVincenzo JP, Whitley RJ, Mackman RL, et al. Oral GS-5806 activity in a respiratory syncytial virus challenge study. N Engl J Med **2014**; 371:711–722.

68. DeVincenzo J, Tait D, Efthimiou J, et al. A Randomized, Placebo-Controlled, Respiratory Syncytial Virus Human Challenge Study of the Antiviral Efficacy, Safety, and Pharmacokinetics of RV521, an Inhibitor of the RSV-F Protein. Antimicrob Agents Chemother **2020**; 64:e01884-19.

69. Deye GA, Miller RS, Miller L, et al. Prolonged protection provided by a single dose of atovaquone-proguanil for the chemoprophylaxis of Plasmodium falciparum malaria in a human challenge model. Clin Infect Dis **2012**; 54:232–239.

70. Dobinson HC, Gibani MM, Jones C, et al. Evaluation of the Clinical and Microbiological Response to Salmonella Paratyphi A Infection in the First Paratyphoid Human Challenge Model. Clin Infect Dis **2017**; 64:1066–1073.

71. Donnenberg MS, Tacket CO, Losonsky G, et al. Effect of prior experimental human enteropathogenic Escherichia coli infection on illness following homologous and heterologous rechallenge. Infect Immun **1998**; 66:52–58.

72. Doyle WJ, Riker DK, McBride TP, et al. Therapeutic effects of an anticholinergic-sympathomimetic combination in induced rhinovirus colds. Ann Otol Rhinol Laryngol **1993**; 102:521–527.

73. Doyle WJ, Skoner DP, Alper CM, et al. Effect of rimantadine treatment on clinical manifestations and otologic complications in adults experimentally infected with influenza A (H1N1) virus. J Infect Dis **1998**; 177:1260–1265.

74. Doyle WJ, Skoner DP, Hayden F, Buchman CA, Seroky JT, Fireman P. Nasal and otologic effects of experimental influenza A virus infection. Ann Otol Rhinol Laryngol **1994**; 103:59–69.

75. Doyle WJ, Gentile DA, Cohen S. Emotional style, nasal cytokines, and illness expression after experimental rhinovirus exposure. Brain Behav Immun **2006**; 20:175–181.

76. Drake CL, Roehrs TA, Royer H, Koshorek G, Turner RB, Roth T. Effects of an experimentally induced rhinovirus cold on sleep, performance, and daytime alertness. Physiol Behav **2000**; 71:75–81.

77. DuPont HL, Chappell CL, Sterling CR, Okhuysen PC, Rose JB, Jakubowski W. The infectivity of Cryptosporidium parvum in healthy volunteers. N Engl J Med **1995**; 332:855–859.

78. Edelman R, Hoffman SL, Davis JR, et al. Long-term persistence of sterile immunity in a volunteer immunized with X-irradiated Plasmodium falciparum sporozoites. J Infect Dis **1993**; 168:1066–1070.

79. Egan JE, Hoffman SL, Haynes JD, et al. Humoral immune responses in volunteers immunized with irradiated Plasmodium falciparum sporozoites. Am J Trop Med Hyg **1993**; 49:166–173.

80. Eiden J, Volckaert B, Rudenko O, et al. 2748. Single Intranasal (IN) Dose of M2SR (M2-Deficient Single Replication) Live Influenza Vaccine Protects Adults Against Subsequent Challenge with a Substantially Drifted H3N2 Strain. Open Forum Infect Dis **2019**; 6:S967–S968.

81. Epstein JE, Tewari K, Lyke KE, et al. Live attenuated malaria vaccine designed to protect through hepatic CD8^+^ T cell immunity. Science **2011**; 334:475–480.

82. Evans DG, Evans DJ, Opekun AR, Graham DY. Non-replicating oral whole cell vaccine protective against enterotoxigenic Escherichia coli (ETEC) diarrhea: stimulation of anti-CFA (CFA/I) and anti-enterotoxin (anti-LT) intestinal IgA and protection against challenge with ETEC belonging to heterologous serotypes. FEMS Microbiol Immunol **1988**; 1:117–125.

83. Feary J, Venn A, Brown A, et al. Safety of hookworm infection in individuals with measurable airway responsiveness: a randomized placebo-controlled feasibility study. Clin Exp Allergy **2009**; 39:1060–1068.

84. Feldman PJ, Cohen S, Doyle WJ, Skoner DP, Gwaltney JM. The impact of personality on the reporting of unfounded symptoms and illness. J Pers Soc Psychol **1999**; 77:370–378.

85. Ferreira DM, Neill DR, Bangert M, et al. Controlled human infection and rechallenge with Streptococcus pneumoniae reveals the protective efficacy of carriage in healthy adults. Am J Respir Crit Care Med **2013**; 187:855–864.

86. Fortney KR, Young RS, Bauer ME, et al. Expression of peptidoglycan-associated lipoprotein is required for virulence in the human model of Haemophilus ducreyi infection. Infect Immun **2000**; 68:6441–6448.

87. Frenck R, Bernstein DI, Xia M, et al. Predicting susceptibility to norovirus GII.4 by use of a challenge model involving humans. J Infect Dis **2012**; 206:1386–1393.

88. Frenck RW, Conti V, Ferruzzi P, et al. Efficacy, safety, and immunogenicity of the Shigella sonnei 1790GAHB GMMA candidate vaccine: Results from a phase 2b randomized, placebo-controlled challenge study in adults. EClinicalMedicine **2021**; 39:101076.

89. Fries LF, Gordon DM, Schneider I, et al. Safety, immunogenicity, and efficacy of a Plasmodium falciparum vaccine comprising a circumsporozoite protein repeat region peptide conjugated to Pseudomonas aeruginosa toxin A. Infect Immun **1992**; 60:1834–1839.

90. Fulcher RA, Cole LE, Janowicz DM, et al. Expression of Haemophilus ducreyi collagen binding outer membrane protein NcaA is required for virulence in swine and human challenge models of chancroid. Infect Immun **2006**; 74:2651–2658.

91. Fullen DJ, Murray B, Mori J, et al. A Tool for Investigating Asthma and COPD Exacerbations: A Newly Manufactured and Well Characterised GMP Wild-Type Human Rhinovirus for Use in the Human Viral Challenge Model. PLoS One **2016**; 11:e0166113.

92. Fullen DJ, Noulin N, Catchpole A, et al. Accelerating Influenza Research: Vaccines, Antivirals, Immunomodulators and Monoclonal Antibodies. The Manufacture of a New Wild-Type H3N2 Virus for the Human Viral Challenge Model. PLoS One **2016**; 11:e0145902.

93. Gentile DA, Fireman P, Skoner DP. Elevations of local leukotriene C4 levels during viral upper respiratory tract infections. Ann Allergy Asthma Immunol **2003**; 91:270–274.

94. Gern JE, Stone CK, Nakano M, et al. Effect of upper respiratory tract infection on AIR inhaled insulin pharmacokinetics and glucodynamics in healthy subjects. Clin Pharmacol Ther **2008**; 83:307–311.

95. Gibani MM, Jin C, Shrestha S, et al. Homologous and heterologous re-challenge with Salmonella Typhi and Salmonella Paratyphi A in a randomised controlled human infection model. PLoS Negl Trop Dis **2020**; 14:e0008783.

96. Gibani MM, Jones E, Barton A, et al. Investigation of the role of typhoid toxin in acute typhoid fever in a human challenge model. Nat Med **2019**; 25:1082–1088.

97. Glennie S, Gritzfeld JF, Pennington SH, et al. Modulation of nasopharyngeal innate defenses by viral coinfection predisposes individuals to experimental pneumococcal carriage. Mucosal Immunol **2016**; 9:56–67.

98. Gómez-Pérez GP, Legarda A, Muñoz J, et al. Controlled human malaria infection by intramuscular and direct venous inoculation of cryopreserved Plasmodium falciparum sporozoites in malaria-naïve volunteers: effect of injection volume and dose on infectivity rates. Malar J **2015**; 14:306.

99. Gordon DM, McGovern TW, Krzych U, et al. Safety, immunogenicity, and efficacy of a recombinantly produced Plasmodium falciparum circumsporozoite protein-hepatitis B surface antigen subunit vaccine. J Infect Dis **1995**; 171:1576–1585.

100. Graham DY, Estes MK, Gentry LO. Double-blind comparison of bismuth subsalicylate and placebo in the prevention and treatment of enterotoxigenic Escherichia coli-induced diarrhea in volunteers. Gastroenterology **1983**; 85:1017–1022.

101. Graham DY, Opekun AR, Osato MS, et al. Challenge model for Helicobacter pylori infection in human volunteers. Gut **2004**; 53:1235–1243.

102. Gritzfeld JF, Cremers AJH, Ferwerda G, et al. Density and duration of experimental human pneumococcal carriage. Clin Microbiol Infect **2014**; 20:O1145-1151.

103. Gritzfeld JF, Wright AD, Collins AM, et al. Experimental human pneumococcal carriage. J Vis Exp **2013**; :50115.

104. Grünberg K, Sharon RF, Sont JK, et al. Rhinovirus-induced airway inflammation in asthma: effect of treatment with inhaled corticosteroids before and during experimental infection. Am J Respir Crit Care Med **2001**; 164:1816–1822.

105. Gubareva LV, Kaiser L, Matrosovich MN, Soo-Hoo Y, Hayden FG. Selection of influenza virus mutants in experimentally infected volunteers treated with oseltamivir. J Infect Dis **2001**; 183:523–531.

106. Gustafson LM, Proud D, Hendley JO, Hayden FG, Gwaltney JM. Oral prednisone therapy in experimental rhinovirus infections. J Allergy Clin Immunol **1996**; 97:1009–1014.

107. Gwaltney JM, Druce HM. Efficacy of brompheniramine maleate for the treatment of rhinovirus colds. Clin Infect Dis **1997**; 25:1188–1194.

108. Gwaltney JM, Park J, Paul RA, Edelman DA, O’Connor RR, Turner RB. Randomized controlled trial of clemastine fumarate for treatment of experimental rhinovirus colds. Clin Infect Dis **1996**; 22:656–662.

109. Gwaltney JM, Winther B, Patrie JT, Hendley JO. Combined antiviral-antimediator treatment for the common cold. J Infect Dis **2002**; 186:147–154.

110. Habibi MS, Jozwik A, Makris S, et al. Impaired Antibody-mediated Protection and Defective IgA B-Cell Memory in Experimental Infection of Adults with Respiratory Syncytial Virus. Am J Respir Crit Care Med **2015**; 191:1040–1049.

111. Hales C, Jochems SP, Robinson R, et al. Symptoms associated with influenza vaccination and experimental human pneumococcal colonisation of the nasopharynx. Vaccine **2020**; 38:2298–2306.

112. Harris JM, Gwaltney JM. Incubation periods of experimental rhinovirus infection and illness. Clin Infect Dis **1996**; 23:1287–1290.

113. Harris SA, Meyer J, Satti I, et al. Evaluation of a human BCG challenge model to assess antimycobacterial immunity induced by BCG and a candidate tuberculosis vaccine, MVA85A, alone and in combination. J Infect Dis **2014**; 209:1259–1268.

114. Harro C, Chakraborty S, Feller A, et al. Refinement of a human challenge model for evaluation of enterotoxigenic Escherichia coli vaccines. Clin Vaccine Immunol **2011**; 18:1719–1727.

115. Harro C, Louis Bourgeois A, Sack D, et al. Live attenuated enterotoxigenic Escherichia coli (ETEC) vaccine with dmLT adjuvant protects human volunteers against virulent experimental ETEC challenge. Vaccine **2019**; 37:1978–1986.

116. Hayden FG, Andries K, Janssen PA. Safety and efficacy of intranasal pirodavir (R77975) in experimental rhinovirus infection. Antimicrob Agents Chemother **1992**; 36:727–732.

117. Hayden FG, Gwaltney JM, Colonno RJ. Modification of experimental rhinovirus colds by receptor blockade. Antiviral Res **1988**; 9:233–247.

118. Hayden FG, Treanor JJ, Betts RF, Lobo M, Esinhart JD, Hussey EK. Safety and efficacy of the neuraminidase inhibitor GG167 in experimental human influenza. JAMA **1996**; 275:295–299.

119. Hayden FG, Treanor JJ, Fritz RS, et al. Use of the oral neuraminidase inhibitor oseltamivir in experimental human influenza: randomized controlled trials for prevention and treatment. JAMA **1999**; 282:1240–1246.

120. Hayden FG, Tunkel AR, Treanor JJ, Betts RF, Allerheiligen S, Harris J. Oral LY217896 for prevention of experimental influenza A virus infection and illness in humans. Antimicrob Agents Chemother **1994**; 38:1178–1181.

121. Hayden FG, Zylidnikov DM, Iljenko VI, Padolka YV. Comparative therapeutic effect of aerosolized and oral rimantadine HCl in experimental human influenza A virus infection. Antiviral Res **1982**; 2:147–153.

122. Healy SA, Murphy SC, Hume JCC, et al. Chemoprophylaxis Vaccination: Phase I Study to Explore Stage-specific Immunity to Plasmodium falciparum in US Adults. Clin Infect Dis **2020**; 71:1481–1490.

123. Herrera S, Fernández O, Manzano MR, et al. Successful sporozoite challenge model in human volunteers with Plasmodium vivax strain derived from human donors. Am J Trop Med Hyg **2009**; 81:740–746.

124. Herrera S, Solarte Y, Jordán-Villegas A, et al. Consistent safety and infectivity in sporozoite challenge model of Plasmodium vivax in malaria-naive human volunteers. Am J Trop Med Hyg **2011**; 84:4–11.

125. Herrington DA, Clyde DF, Davis JR, et al. Human studies with synthetic peptide sporozoite vaccine (NANP)3-TT and immunization with irradiated sporozoites. Bull World Health Organ **1990**; 68 Suppl:33–37.

126. Herrington DA, Clyde DF, Losonsky G, et al. Safety and immunogenicity in man of a synthetic peptide malaria vaccine against Plasmodium falciparum sporozoites. Nature **1987**; 328:257–259.

127. Herrington DA, Van de Verg L, Formal SB, et al. Studies in volunteers to evaluate candidate Shigella vaccines: further experience with a bivalent Salmonella typhi-Shigella sonnei vaccine and protection conferred by previous Shigella sonnei disease. Vaccine **1990**; 8:353–357.

128. Herrington D, Davis J, Nardin E, et al. Successful immunization of humans with irradiated malaria sporozoites: humoral and cellular responses of the protected individuals. Am J Trop Med Hyg **1991**; 45:539–547.

129. Hickey BW, Lumsden JM, Reyes S, et al. Mosquito bite immunization with radiation-attenuated Plasmodium falciparum sporozoites: safety, tolerability, protective efficacy and humoral immunogenicity. Malar J **2016**; 15:377.

130. Higgins PG, al-Nakib W, Barrow GI, Tyrrell DA. Recombinant human interferon-gamma as prophylaxis against rhinovirus colds in volunteers. J Interferon Res **1988**; 8:591–596.

131. Hobbins TE, Hughes TP, Rennels MB, Murphy BR, Levine MM. Bronchial reactivity in experimental infections with influenza virus. J Infect Dis **1982**; 146:468–471.

132. Hodgson SH, Ewer KJ, Bliss CM, et al. Evaluation of the efficacy of ChAd63-MVA vectored vaccines expressing circumsporozoite protein and ME-TRAP against controlled human malaria infection in malaria-naive individuals. J Infect Dis **2015**; 211:1076–1086.

133. Hodgson SH, Juma E, Salim A, et al. Evaluating controlled human malaria infection in Kenyan adults with varying degrees of prior exposure to Plasmodium falciparum using sporozoites administered by intramuscular injection. Front Microbiol **2014**; 5:686.

134. Hoffman SL, Edelman R, Bryan JP, et al. Safety, immunogenicity, and efficacy of a malaria sporozoite vaccine administered with monophosphoryl lipid A, cell wall skeleton of mycobacteria, and squalane as adjuvant. Am J Trop Med Hyg **1994**; 51:603–612.

135. Hoogerwerf M-A, Koopman JPR, Janse JJ, et al. A Randomized Controlled Trial to Investigate Safety and Variability of Egg Excretion After Repeated Controlled Human Hookworm Infection. J Infect Dis **2021**; 223:905–913.

136. Humphreys TL, Li L, Li X, et al. Dysregulated immune profiles for skin and dendritic cells are associated with increased host susceptibility to Haemophilus ducreyi infection in human volunteers. Infect Immun **2007**; 75:5686–5697.

137. Humphreys TL, Schnizlein-Bick CT, Katz BP, et al. Evolution of the cutaneous immune response to experimental Haemophilus ducreyi infection and its relevance to HIV-1 acquisition. J Immunol **2002**; 169:6316–6323.

138. Ishizuka AS, Lyke KE, DeZure A, et al. Protection against malaria at 1 year and immune correlates following PfSPZ vaccination. Nat Med **2016**; 22:614–623.

139. Janowicz D, Leduc I, Fortney KR, Katz BP, Elkins C, Spinola SM. A DltA mutant of Haemophilus ducreyi Is partially attenuated in its ability to cause pustules in human volunteers. Infect Immun **2006**; 74:1394–1397.

140. Janowicz D, Luke NR, Fortney KR, Katz BP, Campagnari AA, Spinola SM. Expression of OmpP2A and OmpP2B is not required for pustule formation by Haemophilus ducreyi in human volunteers. Microb Pathog **2006**; 40:110–115.

141. Janowicz DM, Fortney KR, Katz BP, et al. Expression of the LspA1 and LspA2 proteins by Haemophilus ducreyi is required for virulence in human volunteers. Infect Immun **2004**; 72:4528–4533.

142. Janowicz DM, Tenner-Racz K, Racz P, et al. Experimental infection with Haemophilus ducreyi in persons who are infected with HIV does not cause local or augment systemic viral replication. J Infect Dis **2007**; 195:1443–1451.

143. Jin C, Gibani MM, Moore M, et al. Efficacy and immunogenicity of a Vi-tetanus toxoid conjugate vaccine in the prevention of typhoid fever using a controlled human infection model of Salmonella Typhi: a randomised controlled, phase 2b trial. Lancet **2017**; 390:2472–2480.

144. Jones S, Evans K, McElwaine-Johnn H, et al. DNA vaccination protects against an influenza challenge in a double-blind randomised placebo-controlled phase 1b clinical trial. Vaccine **2009**; 27:2506–2512.

145. Jongo SA, Shekalaghe SA, Church LWP, et al. Safety, Immunogenicity, and Protective Efficacy against Controlled Human Malaria Infection of Plasmodium falciparum Sporozoite Vaccine in Tanzanian Adults. Am J Trop Med Hyg **2018**; 99:338–349.

146. Kapulu MC, Njuguna P, Hamaluba M, et al. Safety and PCR monitoring in 161 semi-immune Kenyan adults following controlled human malaria infection. JCI Insight **2021**; 6:146443.

147. Keitel WA, Couch RB, Cate TR, Six HR, Baxter BD. Cold recombinant influenza B/Texas/1/84 vaccine virus (CRB 87): attenuation, immunogenicity, and efficacy against homotypic challenge. J Infect Dis **1990**; 161:22–26.

148. Kirkpatrick BD, Whitehead SS, Pierce KK, et al. The live attenuated dengue vaccine TV003 elicits complete protection against dengue in a human challenge model. Sci Transl Med **2016**; 8:330ra36.

149. Kotloff KL, Herrington DA, Hale TL, et al. Safety, immunogenicity, and efficacy in monkeys and humans of invasive Escherichia coli K-12 hybrid vaccine candidates expressing Shigella flexneri 2a somatic antigen. Infect Immun **1992**; 60:2218–2224.

150. Kotloff KL, Losonsky GA, Nataro JP, et al. Evaluation of the safety, immunogenicity, and efficacy in healthy adults of four doses of live oral hybrid Escherichia coli-Shigella flexneri 2a vaccine strain EcSf2a-2. Vaccine **1995**; 13:495–502.

151. Kotloff KL, Nataro JP, Losonsky GA, et al. A modified Shigella volunteer challenge model in which the inoculum is administered with bicarbonate buffer: clinical experience and implications for Shigella infectivity. Vaccine **1995**; 13:1488–1494.

152. Krause A, Dingemanse J, Mathis A, Marquart L, Möhrle JJ, McCarthy JS. Pharmacokinetic/pharmacodynamic modelling of the antimalarial effect of Actelion-451840 in an induced blood stage malaria study in healthy subjects. Br J Clin Pharmacol **2016**; 82:412–421.

153. Lambkin-Williams R, Gelder C, Broughton R, et al. An Intranasal Proteosome-Adjuvanted Trivalent Influenza Vaccine Is Safe, Immunogenic & Efficacious in the Human Viral Influenza Challenge Model. Serum IgG & Mucosal IgA Are Important Correlates of Protection against Illness Associated with Infection. PLoS One **2016**; 11:e0163089.

154. Langenberg MCC, Hoogerwerf M-A, Koopman JPR, et al. A controlled human Schistosoma mansoni infection model to advance novel drugs, vaccines and diagnostics. Nat Med **2020**; 26:326–332.

155. Langenberg MCC, Wammes LJ, McCall MBB, et al. Controlled Human Malaria Infection with Graded Numbers of Plasmodium falciparum NF135.C10- or NF166.C8-Infected Mosquitoes. Am J Trop Med Hyg **2018**; 99:709–712.

156. Laurens MB, Billingsley P, Richman A, et al. Successful human infection with P. falciparum using three aseptic Anopheles stephensi mosquitoes: a new model for controlled human malaria infection. PLoS One **2013**; 8:e68969.

157. Leduc I, Banks KE, Fortney KR, et al. Evaluation of the repertoire of the TonB-dependent receptors of Haemophilus ducreyi for their role in virulence in humans. J Infect Dis **2008**; 197:1103–1109.

158. Lell B, Mordmüller B, Dejon Agobe J-C, et al. Impact of Sickle Cell Trait and Naturally Acquired Immunity on Uncomplicated Malaria after Controlled Human Malaria Infection in Adults in Gabon. Am J Trop Med Hyg **2018**; 98:508–515.

159. Lemanske RF, Dick EC, Swenson CA, Vrtis RF, Busse WW. Rhinovirus upper respiratory infection increases airway hyperreactivity and late asthmatic reactions. J Clin Invest **1989**; 83:1–10.

160. Lillie PJ, Duncan CJA, Sheehy SH, et al. Distinguishing malaria and influenza: early clinical features in controlled human experimental infection studies. Travel Med Infect Dis **2012**; 10:192–196.

161. Lindesmith L, Moe C, Marionneau S, et al. Human susceptibility and resistance to Norwalk virus infection. Nat Med **2003**; 9:548–553.

162. Livezey J, Twomey P, Morrison M, et al. An open label study of the safety and efficacy of a single dose of weekly chloroquine and azithromycin administered for malaria prophylaxis in healthy adults challenged with 7G8 chloroquine-resistant Plasmodium falciparum in a controlled human malaria infection model. Malar J **2020**; 19:336.

163. Lyke KE, Ishizuka AS, Berry AA, et al. Attenuated PfSPZ Vaccine induces strain-transcending T cells and durable protection against heterologous controlled human malaria infection. Proc Natl Acad Sci U S A **2017**; 114:2711–2716.

164. Lyke KE, Laurens M, Adams M, et al. Plasmodium falciparum malaria challenge by the bite of aseptic Anopheles stephensi mosquitoes: results of a randomized infectivity trial. PLoS One **2010**; 5:e13490.

165. Lyke KE, Laurens MB, Strauss K, et al. Optimizing Intradermal Administration of Cryopreserved Plasmodium falciparum Sporozoites in Controlled Human Malaria Infection. Am J Trop Med Hyg **2015**; 93:1274–1284.

166. Mackowiak PA, Wasserman SS, Levine MM. An analysis of the quantitative relationship between oral temperature and severity of illness in experimental shigellosis. J Infect Dis **1992**; 166:1181–1184.

167. Mackowiak PA, Wasserman SS, Tacket CO, et al. Quantitative relationship between oral temperature and severity of illness following inoculation with candidate attenuated dengue virus vaccines. Clin Infect Dis **1994**; 19:948–950.

168. Malcolm BA, Aerts CA, Dubois KJ, et al. PrEP-001 prophylactic effect against rhinovirus and influenza virus - RESULTS of 2 randomized trials. Antiviral Res **2018**; 153:70–77.

169. Mallia P, Footitt J, Sotero R, et al. Rhinovirus infection induces degradation of antimicrobial peptides and secondary bacterial infection in chronic obstructive pulmonary disease. Am J Respir Crit Care Med **2012**; 186:1117–1124.

170. Mallia P, Message SD, Kebadze T, Parker HL, Kon OM, Johnston SL. An experimental model of rhinovirus induced chronic obstructive pulmonary disease exacerbations: a pilot study. Respir Res **2006**; 7:116.

171. Mammen MP, Lyons A, Innis BL, et al. Evaluation of dengue virus strains for human challenge studies. Vaccine **2014**; 32:1488–1494.

172. Mateo R, Lindesmith LC, Garg SJ, et al. Production and Clinical Evaluation of Norwalk GI.1 Virus Lot 001-09NV in Norovirus Vaccine Development. J Infect Dis **2020**; 221:919–926.

173. McBride JM, Lim JJ, Burgess T, et al. Phase 2 Randomized Trial of the Safety and Efficacy of MHAA4549A, a Broadly Neutralizing Monoclonal Antibody, in a Human Influenza A Virus Challenge Model. Antimicrob Agents Chemother **2017**; 61:e01154-17.

174. McCall MBB, Wammes LJ, Langenberg MCC, et al. Infectivity of Plasmodium falciparum sporozoites determines emerging parasitemia in infected volunteers. Sci Transl Med **2017**; 9:eaag2490.

175. McCarthy JS, Baker M, O’Rourke P, et al. Efficacy of OZ439 (artefenomel) against early Plasmodium falciparum blood-stage malaria infection in healthy volunteers. J Antimicrob Chemother **2016**; 71:2620–2627.

176. McCarthy JS, Donini C, Chalon S, et al. A Phase 1, Placebo-controlled, Randomized, Single Ascending Dose Study and a Volunteer Infection Study to Characterize the Safety, Pharmacokinetics, and Antimalarial Activity of the Plasmodium Phosphatidylinositol 4-Kinase Inhibitor MMV390048. Clin Infect Dis **2020**; 71:e657–e664.

177. McCarthy JS, Griffin PM, Sekuloski S, et al. Experimentally induced blood-stage Plasmodium vivax infection in healthy volunteers. J Infect Dis **2013**; 208:1688–1694.

178. McCarthy JS, Lotharius J, Rückle T, et al. Safety, tolerability, pharmacokinetics, and activity of the novel long-acting antimalarial DSM265: a two-part first-in-human phase 1a/1b randomised study. Lancet Infect Dis **2017**; 17:626–635.

179. McCarthy JS, Marquart L, Sekuloski S, et al. Linking Murine and Human Plasmodium falciparum Challenge Models in a Translational Path for Antimalarial Drug Development. Antimicrob Agents Chemother **2016**; 60:3669–3675.

180. McCarthy JS, Rückle T, Djeriou E, et al. A Phase II pilot trial to evaluate safety and efficacy of ferroquine against early Plasmodium falciparum in an induced blood-stage malaria infection study. Malar J **2016**; 15:469.

181. McClain MT, Henao R, Williams J, et al. Differential evolution of peripheral cytokine levels in symptomatic and asymptomatic responses to experimental influenza virus challenge. Clin Exp Immunol **2016**; 183:441–451.

182. Memoli MJ, Shaw PA, Han A, et al. Evaluation of Antihemagglutinin and Antineuraminidase Antibodies as Correlates of Protection in an Influenza A/H1N1 Virus Healthy Human Challenge Model. mBio **2016**; 7:e00417-00416.

183. Message SD, Laza-Stanca V, Mallia P, et al. Rhinovirus-induced lower respiratory illness is increased in asthma and related to virus load and Th1/2 cytokine and IL-10 production. Proc Natl Acad Sci U S A **2008**; 105:13562–13567.

184. Minassian AM, Satti I, Poulton ID, Meyer J, Hill AVS, McShane H. A human challenge model for Mycobacterium tuberculosis using Mycobacterium bovis bacille Calmette-Guerin. J Infect Dis **2012**; 205:1035–1042.

185. Minhinnick A, Harris S, Wilkie M, et al. Optimization of a Human Bacille Calmette-Guérin Challenge Model: A Tool to Evaluate Antimycobacterial Immunity. J Infect Dis **2016**; 213:824–830.

186. Mordmüller B, Supan C, Sim KL, et al. Direct venous inoculation of Plasmodium falciparum sporozoites for controlled human malaria infection: a dose-finding trial in two centres. Malar J **2015**; 14:117.

187. Mordmüller B, Surat G, Lagler H, et al. Sterile protection against human malaria by chemoattenuated PfSPZ vaccine. Nature **2017**; 542:445–449.

188. Moss DM, Chappell CL, Okhuysen PC, et al. The antibody response to 27-, 17-, and 15-kDa Cryptosporidium antigens following experimental infection in humans. J Infect Dis **1998**; 178:827–833.

189. Muehling LM, Mai DT, Kwok WW, Heymann PW, Pomés A, Woodfolk JA. Circulating Memory CD4+ T Cells Target Conserved Epitopes of Rhinovirus Capsid Proteins and Respond Rapidly to Experimental Infection in Humans. J Immunol **2016**; 197:3214–3224.

190. Murphy AW, Platts-Mills TA, Lobo M, Hayden F. Respiratory nitric oxide levels in experimental human influenza. Chest **1998**; 114:452–456.

191. Murphy BR, Chanock RM, Clements ML, et al. Evaluation of A/Alaska/6/77 (H3N2) cold-adapted recombinant viruses derived from A/Ann Arbor/6/60 cold-adapted donor virus in adult seronegative volunteers. Infect Immun **1981**; 32:693–697.

192. Murphy SC, Duke ER, Shipman KJ, et al. A Randomized Trial Evaluating the Prophylactic Activity of DSM265 Against Preerythrocytic Plasmodium falciparum Infection During Controlled Human Malarial Infection by Mosquito Bites and Direct Venous Inoculation. J Infect Dis **2018**; 217:693–702.

193. Nash TE, Herrington DA, Levine MM, Conrad JT, Merritt JW. Antigenic variation of Giardia lamblia in experimental human infections. J Immunol **1990**; 144:4362–4369.

194. Nguyen-Van-Tam JS, Killingley B, Enstone J, et al. Minimal transmission in an influenza A (H3N2) human challenge-transmission model within a controlled exposure environment. PLoS Pathog **2020**; 16:e1008704.

195. Noah TL, Becker S. Chemokines in nasal secretions of normal adults experimentally infected with respiratory syncytial virus. Clin Immunol **2000**; 97:43–49.

196. Oxford JS, Schild GC, Corcoran T, et al. A host-cell-selected variant of influenza B virus with a single nucleotide substitution in HA affecting a potential glycosylation site was attenuated in virulence for volunteers. Arch Virol **1990**; 110:37–46.

197. Palmer KL, Thornton AC, Fortney KR, Hood AF, Munson RS, Spinola SM. Evaluation of an isogenic hemolysin-deficient mutant in the human model of Haemophilus ducreyi infection. J Infect Dis **1998**; 178:191–199.

198. Pasay CJ, Rockett R, Sekuloski S, et al. Piperaquine Monotherapy of Drug-Susceptible Plasmodium falciparum Infection Results in Rapid Clearance of Parasitemia but Is Followed by the Appearance of Gametocytemia. J Infect Dis **2016**; 214:105–113.

199. Payne RO, Milne KH, Elias SC, et al. Demonstration of the Blood-Stage Plasmodium falciparum Controlled Human Malaria Infection Model to Assess Efficacy of the P. falciparum Apical Membrane Antigen 1 Vaccine, FMP2.1/AS01. J Infect Dis **2016**; 213:1743–1751.

200. Pennington SH, Pojar S, Mitsi E, et al. Polysaccharide-Specific Memory B Cells Predict Protection against Experimental Human Pneumococcal Carriage. Am J Respir Crit Care Med **2016**; 194:1523–1531.

201. Peterson KM, O’Shea M, Stam W, Mohede ICM, Patrie JT, Hayden FG. Effects of dietary supplementation with conjugated linoleic acid on experimental human rhinovirus infection and illness. Antivir Ther **2009**; 14:33–43.

202. Pitisuttithum P, Cohen MB, Phonrat B, et al. A human volunteer challenge model using frozen bacteria of the new epidemic serotype, V. cholerae O139 in Thai volunteers. Vaccine **2001**; 20:920–925.

203. Pitisuttithum P, Islam D, Chamnanchanunt S, et al. Clinical Trial of an Oral Live Shigella sonnei Vaccine Candidate, WRSS1, in Thai Adults. Clin Vaccine Immunol **2016**; 23:564–575.

204. Pleguezuelos O, James E, Fernandez A, et al. Efficacy of FLU-v, a broad-spectrum influenza vaccine, in a randomized phase IIb human influenza challenge study. NPJ Vaccines **2020**; 5:22.

205. Ramos EL, Mitcham JL, Koller TD, et al. Efficacy and safety of treatment with an anti-m2e monoclonal antibody in experimental human influenza. J Infect Dis **2015**; 211:1038–1044.

206. Reichenberg A, Yirmiya R, Schuld A, et al. Cytokine-associated emotional and cognitive disturbances in humans. Arch Gen Psychiatry **2001**; 58:445–452.

207. Reuling IJ, van de Schans LA, Coffeng LE, et al. A randomized feasibility trial comparing four antimalarial drug regimens to induce Plasmodium falciparum gametocytemia in the controlled human malaria infection model. Elife **2018**; 7:e31549.

208. Reuman PD, Bernstein DI, Keefer MC, Young EC, Sherwood JR, Schiff GM. Efficacy and safety of low dosage amantadine hydrochloride as prophylaxis for influenza A. Antiviral Res **1989**; 11:27–40.

209. Rickman LS, Jones TR, Long GW, et al. Plasmodium falciparum-infected Anopheles stephensi inconsistently transmit malaria to humans. Am J Trop Med Hyg **1990**; 43:441–445.

210. Roestenberg M, Bijker EM, Sim BKL, et al. Controlled human malaria infections by intradermal injection of cryopreserved Plasmodium falciparum sporozoites. Am J Trop Med Hyg **2013**; 88:5–13.

211. Roestenberg M, McCall M, Hopman J, et al. Protection against a malaria challenge by sporozoite inoculation. N Engl J Med **2009**; 361:468–477.

212. Roestenberg M, Teirlinck AC, McCall MBB, et al. Long-term protection against malaria after experimental sporozoite inoculation: an open-label follow-up study. Lancet **2011**; 377:1770–1776.

213. Rylance J, de Steenhuijsen Piters WAA, Mina MJ, et al. Two Randomized Trials of the Effect of Live Attenuated Influenza Vaccine on Pneumococcal Colonization. Am J Respir Crit Care Med **2019**; 199:1160–1163.

214. Sakkestad ST, Steinsland H, Skrede S, et al. Experimental Infection of Human Volunteers with the Heat-Stable Enterotoxin-Producing Enterotoxigenic Escherichia coli Strain TW11681. Pathogens **2019**; 8:E84.

215. Sakkestad ST, Steinsland H, Skrede S, et al. A new human challenge model for testing heat-stable toxin-based vaccine candidates for enterotoxigenic Escherichia coli diarrhea - dose optimization, clinical outcomes, and CD4+ T cell responses. PLoS Negl Trop Dis **2019**; 13:e0007823.

216. Schiff GM, Sherwood JR. Clinical activity of pleconaril in an experimentally induced coxsackievirus A21 respiratory infection. J Infect Dis **2000**; 181:20–26.

217. Schiff GM, Young BC, Stefanovic’ GM, et al. Challenge with rubella virus after loss of detectable vaccine-induced antibody. Rev Infect Dis **1985**; 7 Suppl 1:S157-163.

218. Sears SD, Clements ML. Protective efficacy of low-dose amantadine in adults challenged with wild-type influenza A virus. Antimicrob Agents Chemother **1987**; 31:1470–1473.

219. Seder RA, Chang L-J, Enama ME, et al. Protection against malaria by intravenous immunization with a nonreplicating sporozoite vaccine. Science **2013**; 341:1359–1365.

220. Seitz SR, Leon JS, Schwab KJ, et al. Norovirus infectivity in humans and persistence in water. Appl Environ Microbiol **2011**; 77:6884–6888.

221. Sheehy SH, Spencer AJ, Douglas AD, et al. Optimising Controlled Human Malaria Infection Studies Using Cryopreserved P. falciparum Parasites Administered by Needle and Syringe. PLoS One **2013**; 8:e65960.

222. Shekalaghe S, Rutaihwa M, Billingsley PF, et al. Controlled human malaria infection of Tanzanians by intradermal injection of aseptic, purified, cryopreserved Plasmodium falciparum sporozoites. Am J Trop Med Hyg **2014**; 91:471–480.

223. Shmuklarsky MJ, Boudreau EF, Pang LW, et al. Failure of doxycycline as a causal prophylactic agent against Plasmodium falciparum malaria in healthy nonimmune volunteers. Ann Intern Med **1994**; 120:294–299.

224. Sinha A, Lutter R, Xu B, et al. Loss of adaptive capacity in asthmatic patients revealed by biomarker fluctuation dynamics after rhinovirus challenge. Elife **2019**; 8:e47969.

225. Skrede S, Steinsland H, Sommerfelt H, et al. Experimental infection of healthy volunteers with enterotoxigenic Escherichia coli wild-type strain TW10598 in a hospital ward. BMC Infect Dis **2014**; 14:482.

226. Smith CM, Jerkovic A, Truong TT, Foote SJ, McCarthy JS, McMorran BJ. Griseofulvin impairs intraerythrocytic growth of Plasmodium falciparum through ferrochelatase inhibition but lacks activity in an experimental human infection study. Sci Rep **2017**; 7:41975.

227. Snyder MH, Betts RF, DeBorde D, et al. Four viral genes independently contribute to attenuation of live influenza A/Ann Arbor/6/60 (H2N2) cold-adapted reassortant virus vaccines. J Virol **1988**; 62:488–495.

228. Snyder MH, Clements ML, Betts RF, et al. Evaluation of live avian-human reassortant influenza A H3N2 and H1N1 virus vaccines in seronegative adult volunteers. J Clin Microbiol **1986**; 23:852–857.

229. Snyder MH, Clements ML, De Borde D, Maassab HF, Murphy BR. Attenuation of wild-type human influenza A virus by acquisition of the PA polymerase and matrix protein genes of influenza A/Ann Arbor/6/60 cold-adapted donor virus. J Clin Microbiol **1985**; 22:719–725.

230. Sperber SJ, Doyle WJ, McBride TP, Sorrentino JV, Riker DK, Hayden FG. Otologic effects of interferon beta serine in experimental rhinovirus colds. Arch Otolaryngol Head Neck Surg **1992**; 118:933–936.

231. Sperber SJ, Hendley JO, Hayden FG, Riker DK, Sorrentino JV, Gwaltney JM. Effects of naproxen on experimental rhinovirus colds. A randomized, double-blind, controlled trial. Ann Intern Med **1992**; 117:37–41.

232. Sperber SJ, Shah LP, Gilbert RD, Ritchey TW, Monto AS. Echinacea purpurea for prevention of experimental rhinovirus colds. Clin Infect Dis **2004**; 38:1367–1371.

233. Spinola SM, Wild LM, Apicella MA, Gaspari AA, Campagnari AA. Experimental human infection with Haemophilus ducreyi. J Infect Dis **1994**; 169:1146–1150.

234. Spinola SM, Bong CTH, Faber AL, et al. Differences in host susceptibility to disease progression in the human challenge model of Haemophilus ducreyi infection. Infect Immun **2003**; 71:6658–6663.

235. Spinola SM, Fortney KR, Katz BP, et al. Haemophilus ducreyi requires an intact flp gene cluster for virulence in humans. Infect Immun **2003**; 71:7178–7182.

236. Spring MD, Cummings JF, Ockenhouse CF, et al. Phase 1/2a study of the malaria vaccine candidate apical membrane antigen-1 (AMA-1) administered in adjuvant system AS01B or AS02A. PLoS One **2009**; 4:e5254.

237. Stevens M, Rusch S, DeVincenzo J, et al. Antiviral Activity of Oral JNJ-53718678 in Healthy Adult Volunteers Challenged With Respiratory Syncytial Virus: A Placebo-Controlled Study. J Infect Dis **2018**; 218:748–756.

238. Sulyok M, Rückle T, Roth A, et al. DSM265 for Plasmodium falciparum chemoprophylaxis: a randomised, double blinded, phase 1 trial with controlled human malaria infection. Lancet Infect Dis **2017**; 17:636–644.

239. Suntharasamai P, Migasena S, Vongsthongsri U, et al. Clinical and bacteriological studies of El Tor cholera after ingestion of known inocula in Thai volunteers. Vaccine **1992**; 10:502–505.

240. Tacket CO, Binion SB, Bostwick E, Losonsky G, Roy MJ, Edelman R. Efficacy of bovine milk immunoglobulin concentrate in preventing illness after Shigella flexneri challenge. Am J Trop Med Hyg **1992**; 47:276–283.

241. Tacket CO, Cohen MB, Wasserman SS, et al. Randomized, double-blind, placebo-controlled, multicentered trial of the efficacy of a single dose of live oral cholera vaccine CVD 103-HgR in preventing cholera following challenge with Vibrio cholerae O1 El tor inaba three months after vaccination. Infect Immun **1999**; 67:6341–6345.

242. Tacket CO, Forrest B, Morona R, et al. Safety, immunogenicity, and efficacy against cholera challenge in humans of a typhoid-cholera hybrid vaccine derived from Salmonella typhi Ty21a. Infect Immun **1990**; 58:1620–1627.

243. Talaat KR, Porter CK, Bourgeois AL, et al. Oral delivery of Hyperimmune bovine serum antibodies against CS6-expressing enterotoxigenic Escherichia coli as a prophylactic against diarrhea. Gut Microbes **2020**; 12:1732852.

244. Talaat KR, Alaimo C, Martin P, et al. Human challenge study with a Shigella bioconjugate vaccine: Analyses of clinical efficacy and correlate of protection. EBioMedicine **2021**; 66:103310.

245. Talaat KR, Porter CK, Jaep KM, et al. Refinement of the CS6-expressing enterotoxigenic Escherichia coli strain B7A human challenge model: A randomized trial. PLoS One **2020**; 15:e0239888.

246. Talley AK, Healy SA, Finney OC, et al. Safety and comparability of controlled human Plasmodium falciparum infection by mosquito bite in malaria-naïve subjects at a new facility for sporozoite challenge. PLoS One **2014**; 9:e109654.

247. Taylor DN, McKenzie R, Durbin A, et al. Rifaximin, a nonabsorbed oral antibiotic, prevents shigellosis after experimental challenge. Clin Infect Dis **2006**; 42:1283–1288.

248. Teirlinck AC, Roestenberg M, van de Vegte-Bolmer M, et al. NF135.C10: a new Plasmodium falciparum clone for controlled human malaria infections. J Infect Dis **2013**; 207:656–660.

249. Thornton AC, O’Mara EM, Sorensen SJ, et al. Prevention of experimental Haemophilus ducreyi infection: a randomized, controlled clinical trial. J Infect Dis **1998**; 177:1608–1613.

250. Throm RE, Al-Tawfiq JA, Fortney KR, et al. Evaluation of an isogenic major outer membrane protein-deficient mutant in the human model of Haemophilus ducreyi infection. Infect Immun **2000**; 68:2602–2607.

251. Tian SF, Zhang LX, Murphy BR. Characterization of the genotype and level of attenuation of an influenza A reassortant virus produced by mating the Xia-ts donor virus with A/Beijing/70 (H1N1) wild type virus. Vaccine **1984**; 2:189–192.

252. Tribble DR, Baqar S, Carmolli MP, et al. Campylobacter jejuni strain CG8421: a refined model for the study of Campylobacteriosis and evaluation of Campylobacter vaccines in human subjects. Clin Infect Dis **2009**; 49:1512–1519.

253. Tribble DR, Baqar S, Scott DA, et al. Assessment of the duration of protection in Campylobacter jejuni experimental infection in humans. Infect Immun **2010**; 78:1750–1759.

254. Turner RB. Ineffectiveness of intranasal zinc gluconate for prevention of experimental rhinovirus colds. Clin Infect Dis **2001**; 33:1865–1870.

255. Turner RB, Cetnarowski WE. Effect of treatment with zinc gluconate or zinc acetate on experimental and natural colds. Clin Infect Dis **2000**; 31:1202–1208.

256. Turner RB, Dutko FJ, Goldstein NH, Lockwood G, Hayden FG. Efficacy of oral WIN 54954 for prophylaxis of experimental rhinovirus infection. Antimicrob Agents Chemother **1993**; 37:297–300.

257. Turner RB, Riker DK, Gangemi JD. Ineffectiveness of echinacea for prevention of experimental rhinovirus colds. Antimicrob Agents Chemother **2000**; 44:1708–1709.

258. Turner RB, Wecker MT, Pohl G, et al. Efficacy of tremacamra, a soluble intercellular adhesion molecule 1, for experimental rhinovirus infection: a randomized clinical trial. JAMA **1999**; 281:1797–1804.

259. Turner RB, Winther B, Hendley JO, Mygind N, Gwaltney JM. Sites of virus recovery and antigen detection in epithelial cells during experimental rhinovirus infection. Acta Otolaryngol Suppl **1984**; 413:9–14.

260. Turner RB, Bauer R, Woelkart K, Hulsey TC, Gangemi JD. An evaluation of Echinacea angustifolia in experimental rhinovirus infections. N Engl J Med **2005**; 353:341–348.

261. Tyrrell DA, Cohen S, Schlarb JE. Signs and symptoms in common colds. Epidemiol Infect **1993**; 111:143–156.

262. van Hoffen E, Mercenier A, Vidal K, et al. Characterization of the pathophysiological determinants of diarrheagenic Escherichia coli infection using a challenge model in healthy adults. Sci Rep **2021**; 11:6060.

263. Waddington CS, Darton TC, Jones C, et al. An outpatient, ambulant-design, controlled human infection model using escalating doses of Salmonella Typhi challenge delivered in sodium bicarbonate solution. Clin Infect Dis **2014**; 58:1230–1240.

264. Walk J, de Bree LCJ, Graumans W, et al. Outcomes of controlled human malaria infection after BCG vaccination. Nat Commun **2019**; 10:874.

265. Walker JB, Hussey EK, Treanor JJ, Montalvo A, Hayden FG. Effects of the neuraminidase inhibitor zanamavir on otologic manifestations of experimental human influenza. J Infect Dis **1997**; 176:1417–1422.

266. Watson JM, Francis JN, Mesens S, et al. Characterisation of a wild-type influenza (A/H1N1) virus strain as an experimental challenge agent in humans. Virol J **2015**; 12:13.

267. Watts RE, Odedra A, Marquart L, et al. Safety and parasite clearance of artemisinin-resistant Plasmodium falciparum infection: A pilot and a randomised volunteer infection study in Australia. PLoS Med **2020**; 17:e1003203.

268. Weidner TG, Anderson BN, Kaminsky LA, Dick EC, Schurr T. Effect of a rhinovirus-caused upper respiratory illness on pulmonary function test and exercise responses. Med Sci Sports Exerc **1997**; 29:604–609.

269. Wilkinson TM, Li CKF, Chui CSC, et al. Preexisting influenza-specific CD4+ T cells correlate with disease protection against influenza challenge in humans. Nat Med **2012**; 18:274–280.

270. Winokur PL, Chaloner K, Doern GV, Ferreira J, Apicella MA. Safety and immunological outcomes following human inoculation with nontypeable Haemophilus influenzae. J Infect Dis **2013**; 208:728–738.

271. Winther B, Buchert D, Turner RB, Hendley JO, Tschaikin M. Decreased rhinovirus shedding after intranasal oxymetazoline application in adults with induced colds compared with intranasal saline. Am J Rhinol Allergy **2010**; 24:374–377.

272. Wright AKA, Ferreira DM, Gritzfeld JF, et al. Human nasal challenge with Streptococcus pneumoniae is immunising in the absence of carriage. PLoS Pathog **2012**; 8:e1002622.

273. Young RS, Filiatrault MJ, Fortney KR, et al. Haemophilus ducreyi lipooligosaccharide mutant defective in expression of beta-1,4-glucosyltransferase is virulent in humans. Infect Immun **2001**; 69:4180–4184.

274. Young RS, Fortney K, Haley JC, et al. Expression of sialylated or paragloboside-like lipooligosaccharides are not required for pustule formation by Haemophilus ducreyi in human volunteers. Infect Immun **1999**; 67:6335–6340.

275. Young RS, Fortney KR, Gelfanova V, et al. Expression of cytolethal distending toxin and hemolysin is not required for pustule formation by Haemophilus ducreyi in human volunteers. Infect Immun **2001**; 69:1938–1942.

276. Zuckerman MA, Cox RJ, Oxford JS. Attenuation of virulence in influenza B viral infection of volunteers. J Infect **1994**; 28:41–48.

## Reference List of Excluded Studies

The following is a list of articles describing excluded studies, including studies that performed secondary analysis of results from previously conducted HCTs (excluded in favor of including the original articles describing these previously conducted HCTs), as well as articles that reported results from studies described in other articles which had already been included. Reasons for exclusion for all excluded articles are provided in the online dataset.

1. Atmar RL, Bernstein DI, Lyon GM, et al. Serological Correlates of Protection against a GII.4 Norovirus. Clinical and Vaccine Immunology **2015**; 22:923–929.

2. Banks KE, Humphreys TL, Li W, Katz BP, Wilkes DS, Spinola SM. Haemophilus ducreyi Partially Activates Human Myeloid Dendritic Cells. Infection and Immunity **2007**; 75:5678–5685.

3. Bauer ME, Spinola SM. Localization of Haemophilus ducreyi at the Pustular Stage of Disease in the Human Model of Infection. Infection and Immunity **2000**; 68:2309–2314.

4. Bennett JW, Pybus BS, Yadava A, et al. Primaquine Failure and Cytochrome P-450 2D6 in Plasmodium vivax Malaria. http://dx.doi.org/10.1056/NEJMc1301936. 2013; Available at:<https://www.nejm.org/doi/10.1056/NEJMc1301936>. Accessed 7 December 2021.

5. Bong CTH, Harezlak J, Katz BP, Spinola SM. Men Are More Susceptible Than Women to Pustule Formation in the Experimental Model of Haemophilus ducreyi Infection. Sexually Transmitted Diseases **2002**; 29:114–118.

6. Church LWP, Le TP, Bryan JP, et al. Clinical Manifestations of Plasmodium falciparum Malaria Experimentally Induced by Mosquito Challenge. The Journal of Infectious Diseases **1997**; 175:915–920.

7. Clements ML, Betts RF, Tierney EL, Murphy BR. Serum and nasal wash antibodies associated with resistance to experimental challenge with influenza A wild-type virus. Journal of Clinical Microbiology **1986**; 24:157–160.

8. Cohen S, Tyrrell DA, Smith AP. Psychological stress and susceptibility to the common cold. N Engl J Med **1991**; 325:606–612.

9. Doyle WJ, Alper CM, Buchman CA, Moody SA, Skoner DP, Cohen S. Illness and Otological Changes During Upper Respiratory Virus Infection. The Laryngoscope **1999**; 109:324–328.

10. Ducarmon QR, Hoogerwerf MA, Janse JJ, et al. Dynamics of the bacterial gut microbiota during controlled human infection with Necator americanus larvae. Gut Microbes **2020**; 12:1840764.

11. Friedman-Klabanoff DJ, Laurens MB, Berry AA, et al. The Controlled Human Malaria Infection Experience at the University of Maryland. The American Journal of Tropical Medicine and Hygiene **2019**; 100:556–565.

12. Gelfanova V, Humphreys TL, Spinola SM. Characterization of Haemophilus ducreyi-Specific T-Cell Lines from Lesions of Experimentally Infected Human Subjects. Infection and Immunity **2001**; 69:4224–4231.

13. Gwaltney JM Jr, Hendley JO, Patrie JT. Symptom Severity Patterns in Experimental Common Colds and Their Usefulness in Timing Onset of Illness in Natural Colds. Clinical Infectious Diseases **2003**; 36:714–723.

14. Hewson CA, Haas JJ, Bartlett NW, et al. Rhinovirus induces MUC5AC in a human infection model and in vitro via NF-κB and EGFR pathways. European Respiratory Journal **2010**; 36:1425–1435.

15. Hodgson SH, Juma E, Salim A, et al. Lessons learnt from the first controlled human malaria infection study conducted in Nairobi, Kenya. Malaria Journal **2015**; 14:182.

16. Hoffman SL, Goh LML, Luke TC, et al. Protection of Humans against Malaria by Immunization with Radiation-Attenuated Plasmodium falciparum Sporozoites. The Journal of Infectious Diseases **2002**; 185:1155–1164.

17. Janicki-Deverts D, Cohen S, Doyle WJ, Turner RB, Treanor JJ. Infection-induced proinflammatory cytokines are associated with decreases in positive affect, but not increases in negative affect. Brain Behav Immun **2007**; 21:301–307.

18. Janowicz DM, Ofner S, Katz BP, Spinola SM. Experimental Infection of Human Volunteers with Haemophilus ducreyi: Fifteen Years of Clinical Data and Experience. The Journal of Infectious Diseases **2009**; 199:1671–1679.

19. Jin C, Gibani MM, Pennington SH, et al. Treatment responses to Azithromycin and Ciprofloxacin in uncomplicated Salmonella Typhi infection: A comparison of Clinical and Microbiological Data from a Controlled Human Infection Model. PLOS Neglected Tropical Diseases **2019**; 13:e0007955.

20. Lamsfus Calle C, Fendel R, Singh A, et al. Expansion of Functional Myeloid-Derived Suppressor Cells in Controlled Human Malaria Infection. Frontiers in Immunology **2021**; 12:690.

21. Matsumiya M, Satti I, Chomka A, et al. Gene Expression and Cytokine Profile Correlate With Mycobacterial Growth in a Human BCG Challenge Model. The Journal of Infectious Diseases **2015**; 211:1499–1509.

22. Mayo-Smith LM, Simon JK, Chen WH, et al. The Live Attenuated Cholera Vaccine CVD 103-HgR Primes Responses to the Toxin-Coregulated Pilus Antigen TcpA in Subjects Challenged with Wild-Type Vibrio cholerae. Clinical and Vaccine Immunology 24:e00470-16.

23. Medema GJ, Teunis PFM, Havelaar AH, Haas CN. Assessment of the dose-response relationship of Campylobacter jejuni. International Journal of Food Microbiology **1996**; 30:101–111.

24. Mitsi E, Roche AM, Reiné J, et al. Agglutination by anti-capsular polysaccharide antibody is associated with protection against experimental human pneumococcal carriage. Mucosal Immunol **2017**; 10:385–394.

25. Palmer KL, Schnizlein-Bick CT, Orazi A, et al. The Immune Response to Haemophilus ducreyi Resembles a Delayed-Type Hypersensitivity Reaction throughout Experimental Infection of Human Subjects. The Journal of Infectious Diseases **1998**; 178:1688–1697.

26. Plaisance KI, Kudaravalli S, Wasserman SS, Levine MM, Mackowiak PA. Effect of Antipyretic Therapy on the Duration of Illness in Experimental Influenza A, Shigella sonnei, and Rickettsia rickettsii Infections. Pharmacotherapy: The Journal of Human Pharmacology and Drug Therapy **2000**; 20:1417–1422.

27. Porter CK, Lynen A, Riddle MS, et al. Clinical endpoints in the controlled human challenge model for Shigella: A call for standardization and the development of a disease severity score. PLOS ONE **2018**; 13:e0194325.

28. Reeck A, Kavanagh O, Estes MK, et al. Serological Correlate of Protection against Norovirus-Induced Gastroenteritis. The Journal of Infectious Diseases **2010**; 202:1212–1218.

29. Roestenberg M, O’Hara GA, Duncan CJA, et al. Comparison of Clinical and Parasitological Data from Controlled Human Malaria Infection Trials. PLOS ONE **2012**; 7:e38434.

30. Shimanovich AA, Buskirk AD, Heine SJ, et al. Functional and Antigen-Specific Serum Antibody Levels as Correlates of Protection against Shigellosis in a Controlled Human Challenge Study. Clinical and Vaccine Immunology 24:e00412-16.

31. Vallejo AF, García J, Amado-Garavito AB, Arévalo-Herrera M, Herrera S. Plasmodium vivax gametocyte infectivity in sub-microscopic infections. Malaria Journal **2016**; 15:48.

32. Van de Verg L, Herrington DA, Murphy JR, Wasserman SS, Formal SB, Levine MM. Specific immunoglobulin A-secreting cells in peripheral blood of humans following oral immunization with a bivalent Salmonella typhi-Shigella sonnei vaccine or infection by pathogenic S. sonnei. Infection and Immunity **1990**; 58:2002–2004.

33. van Wolfswinkel ME, Langenberg MCC, Wammes LJ, et al. Changes in total and differential leukocyte counts during the clinically silent liver phase in a controlled human malaria infection in malaria-naïve Dutch volunteers. Malaria Journal **2017**; 16:457.

34. Walk J, Schats R, Langenberg MCC, et al. Diagnosis and treatment based on quantitative PCR after controlled human malaria infection. Malaria Journal **2016**; 15:398.

35. Zhu J, Message SD, Qiu Y, et al. Airway Inflammation and Illness Severity in Response to Experimental Rhinovirus Infection in Asthma. CHEST **2014**; 145:1219–1229.

1. Corresponding Author: David Manheim, david@1daysooner.org [↑](#footnote-ref-0)
