# Supplementary material for: A Systematic Review of Human Challenge Trials, Designs, and Safety
Source: Clin Infect Dis. 2022 Oct 11;76(4):609–19. doi: 10.1093/cid/ciac820 (PMC9938741; doi:10.1093/cid/ciac820)
Supplement: ciac820_Supplementary_Data [file ciac820_supplementary_data.zip › Supplementary Material - Protocol.pptx]

## Slide 1
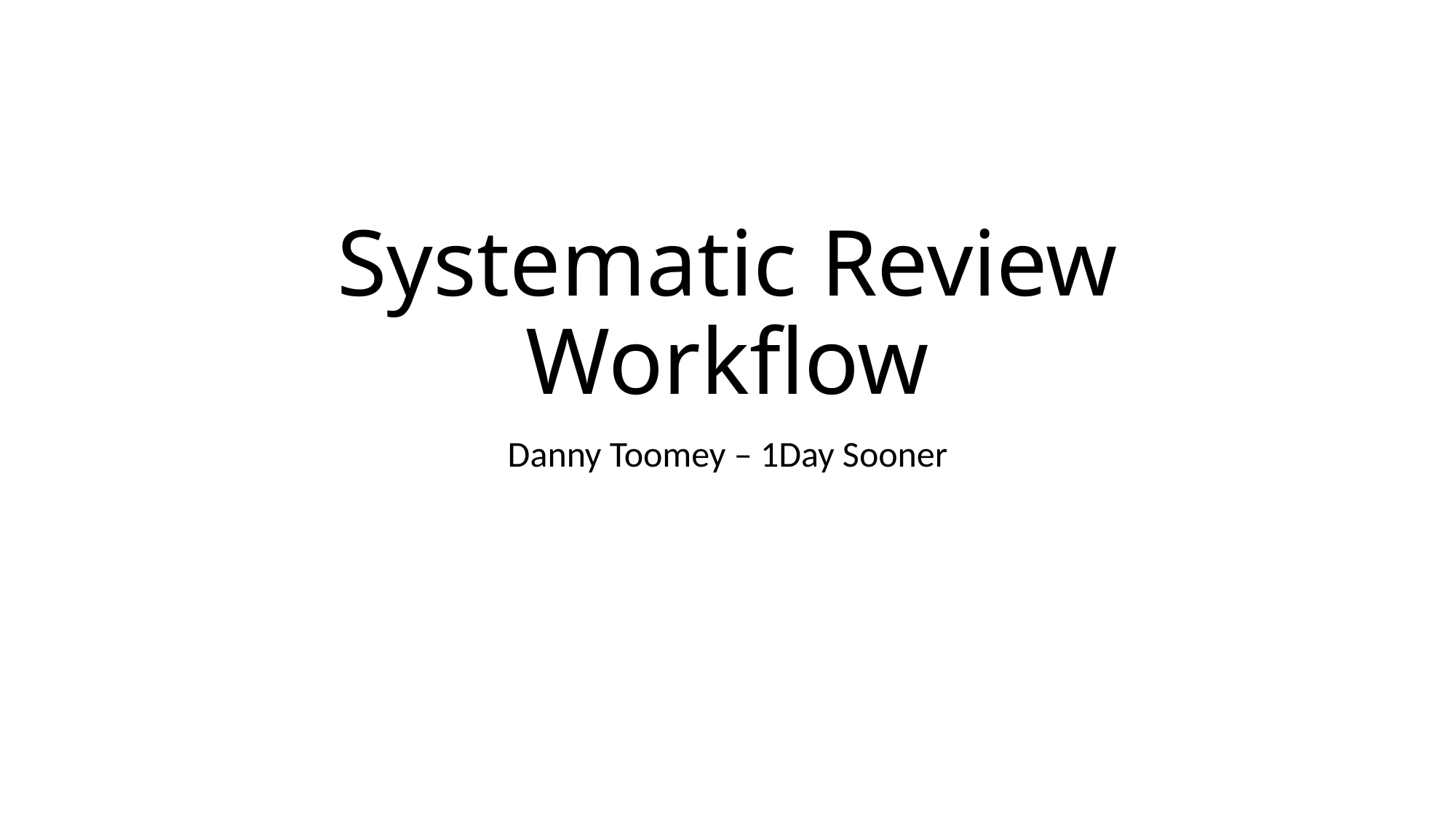

# Systematic Review Workflow
Danny Toomey – 1Day Sooner

## Slide 2
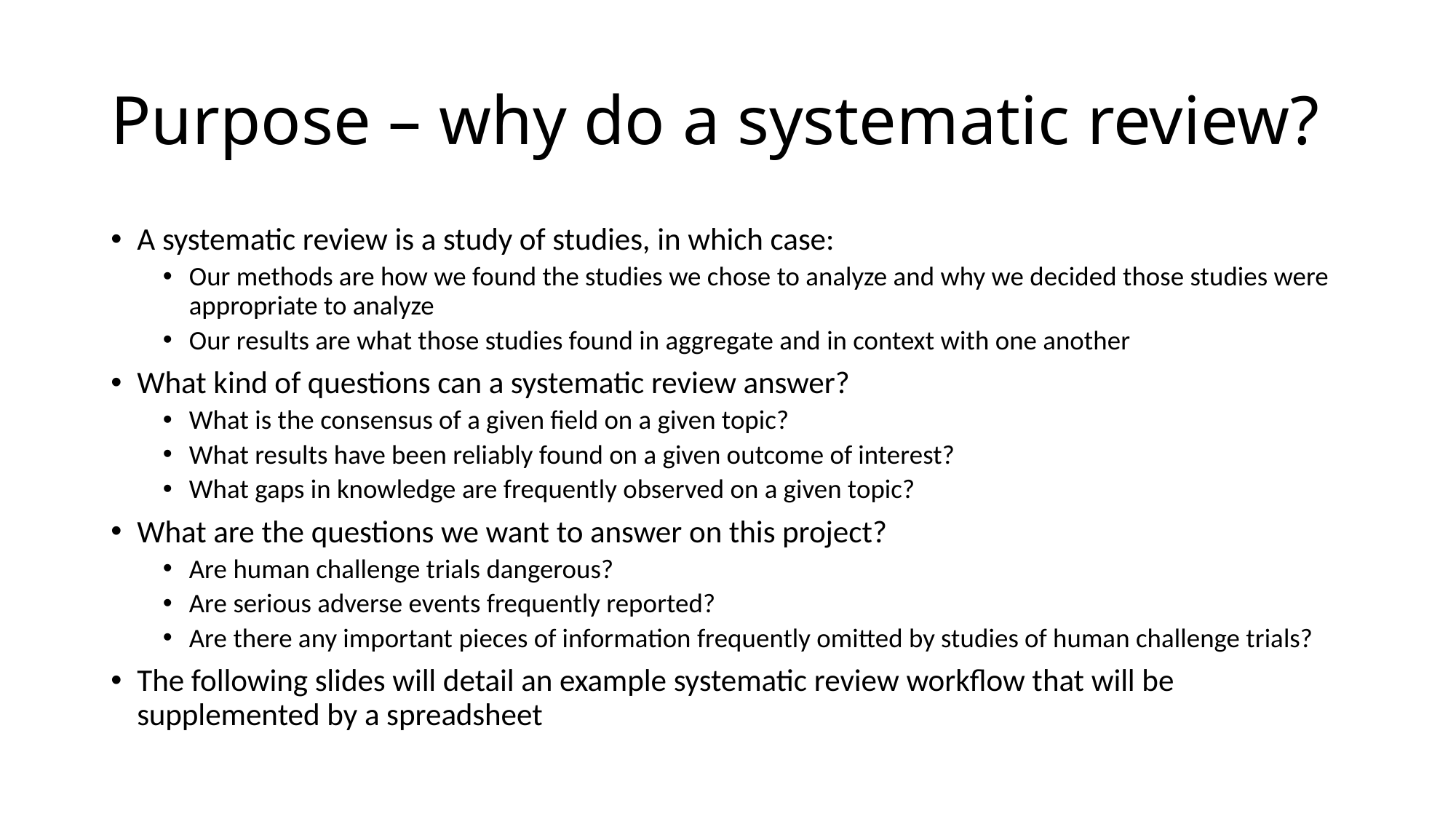

# Purpose – why do a systematic review?
A systematic review is a study of studies, in which case:
Our methods are how we found the studies we chose to analyze and why we decided those studies were appropriate to analyze
Our results are what those studies found in aggregate and in context with one another
What kind of questions can a systematic review answer?
What is the consensus of a given field on a given topic?
What results have been reliably found on a given outcome of interest?
What gaps in knowledge are frequently observed on a given topic?
What are the questions we want to answer on this project?
Are human challenge trials dangerous?
Are serious adverse events frequently reported?
Are there any important pieces of information frequently omitted by studies of human challenge trials?
The following slides will detail an example systematic review workflow that will be supplemented by a spreadsheet

## Slide 3
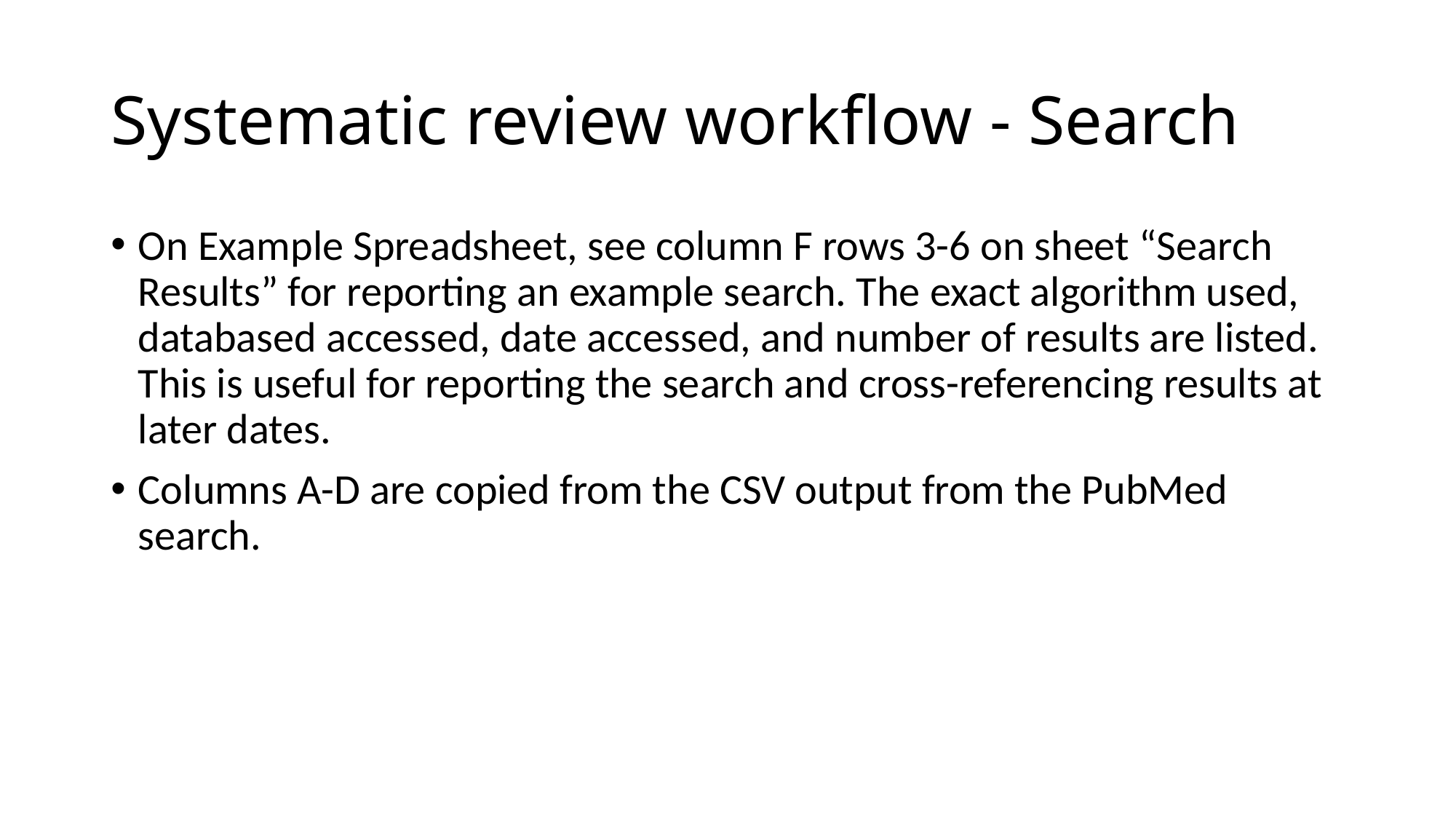

# Systematic review workflow - Search
On Example Spreadsheet, see column F rows 3-6 on sheet “Search Results” for reporting an example search. The exact algorithm used, databased accessed, date accessed, and number of results are listed. This is useful for reporting the search and cross-referencing results at later dates.
Columns A-D are copied from the CSV output from the PubMed search.

## Slide 4
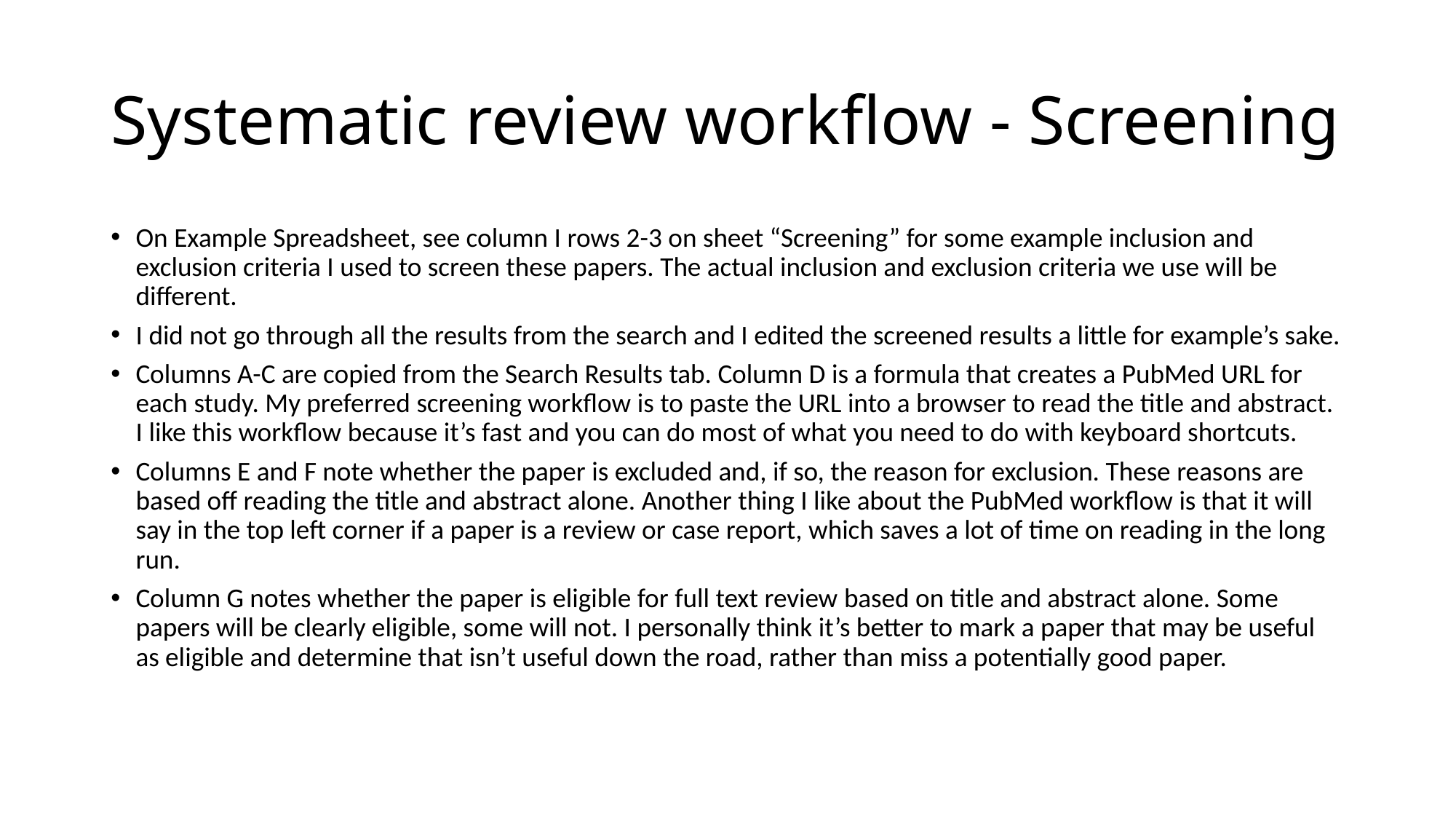

# Systematic review workflow - Screening
On Example Spreadsheet, see column I rows 2-3 on sheet “Screening” for some example inclusion and exclusion criteria I used to screen these papers. The actual inclusion and exclusion criteria we use will be different.
I did not go through all the results from the search and I edited the screened results a little for example’s sake.
Columns A-C are copied from the Search Results tab. Column D is a formula that creates a PubMed URL for each study. My preferred screening workflow is to paste the URL into a browser to read the title and abstract. I like this workflow because it’s fast and you can do most of what you need to do with keyboard shortcuts.
Columns E and F note whether the paper is excluded and, if so, the reason for exclusion. These reasons are based off reading the title and abstract alone. Another thing I like about the PubMed workflow is that it will say in the top left corner if a paper is a review or case report, which saves a lot of time on reading in the long run.
Column G notes whether the paper is eligible for full text review based on title and abstract alone. Some papers will be clearly eligible, some will not. I personally think it’s better to mark a paper that may be useful as eligible and determine that isn’t useful down the road, rather than miss a potentially good paper.

## Slide 5
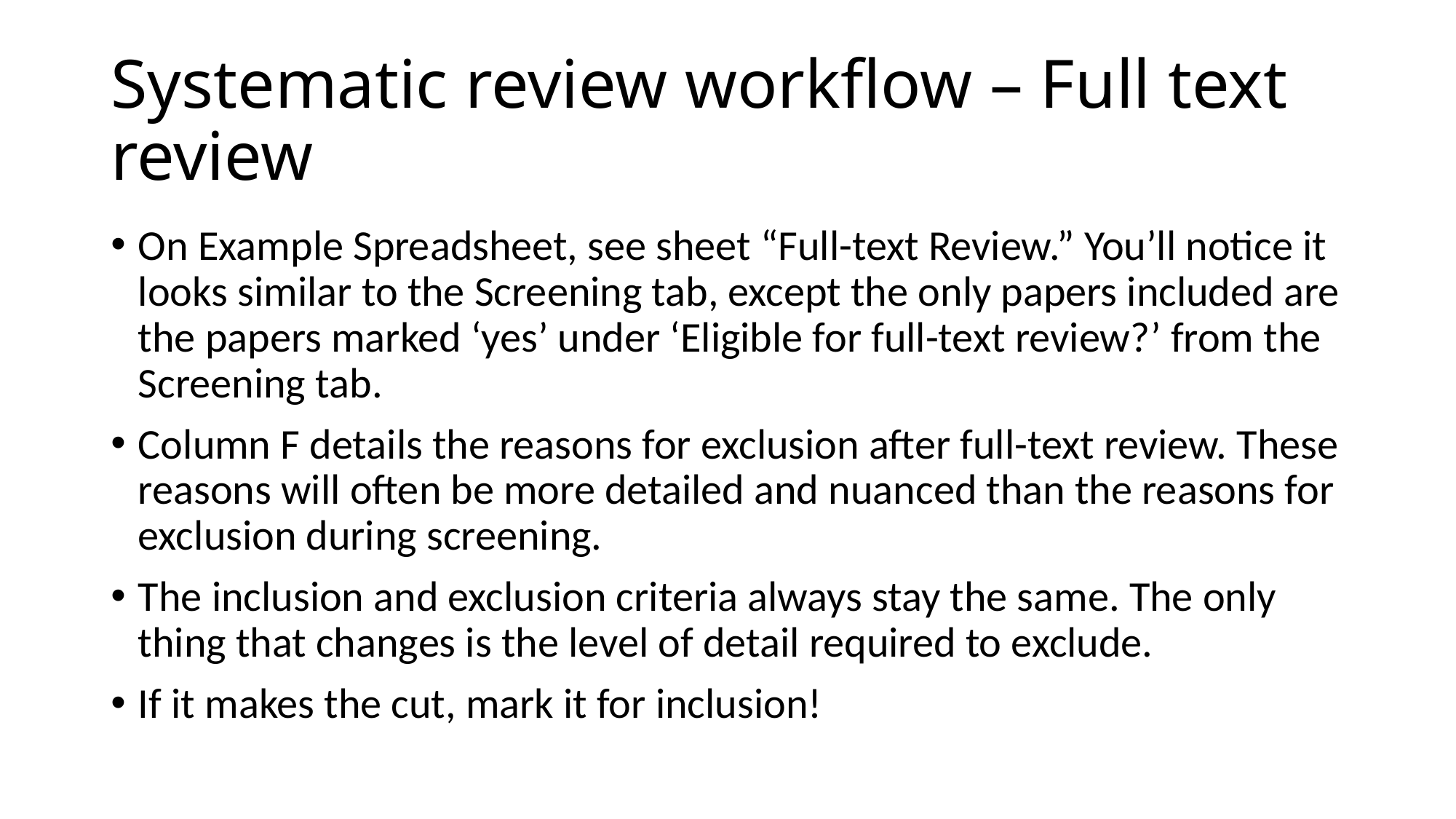

# Systematic review workflow – Full text review
On Example Spreadsheet, see sheet “Full-text Review.” You’ll notice it looks similar to the Screening tab, except the only papers included are the papers marked ‘yes’ under ‘Eligible for full-text review?’ from the Screening tab.
Column F details the reasons for exclusion after full-text review. These reasons will often be more detailed and nuanced than the reasons for exclusion during screening.
The inclusion and exclusion criteria always stay the same. The only thing that changes is the level of detail required to exclude.
If it makes the cut, mark it for inclusion!

## Slide 6
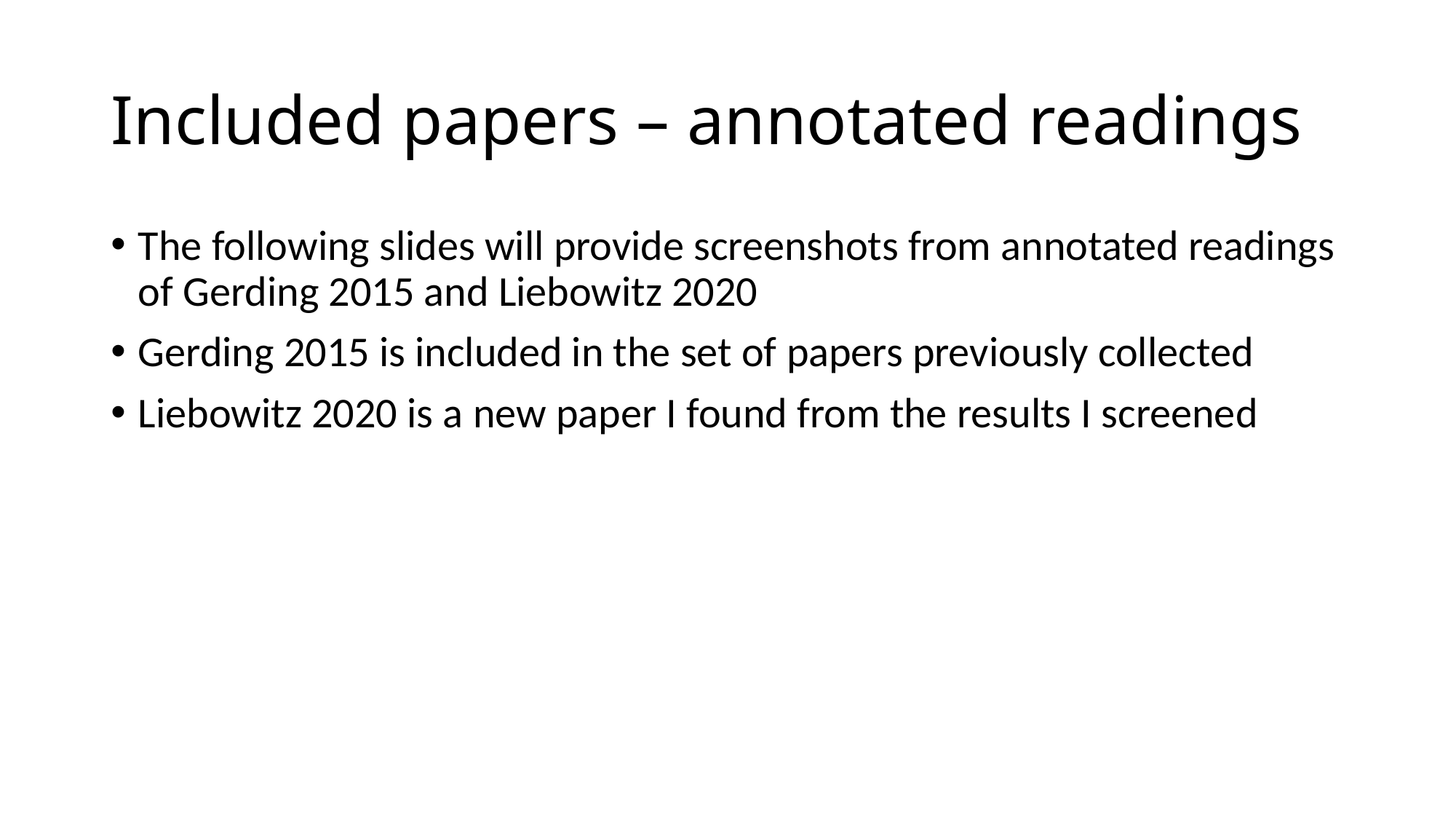

# Included papers – annotated readings
The following slides will provide screenshots from annotated readings of Gerding 2015 and Liebowitz 2020
Gerding 2015 is included in the set of papers previously collected
Liebowitz 2020 is a new paper I found from the results I screened

## Slide 7
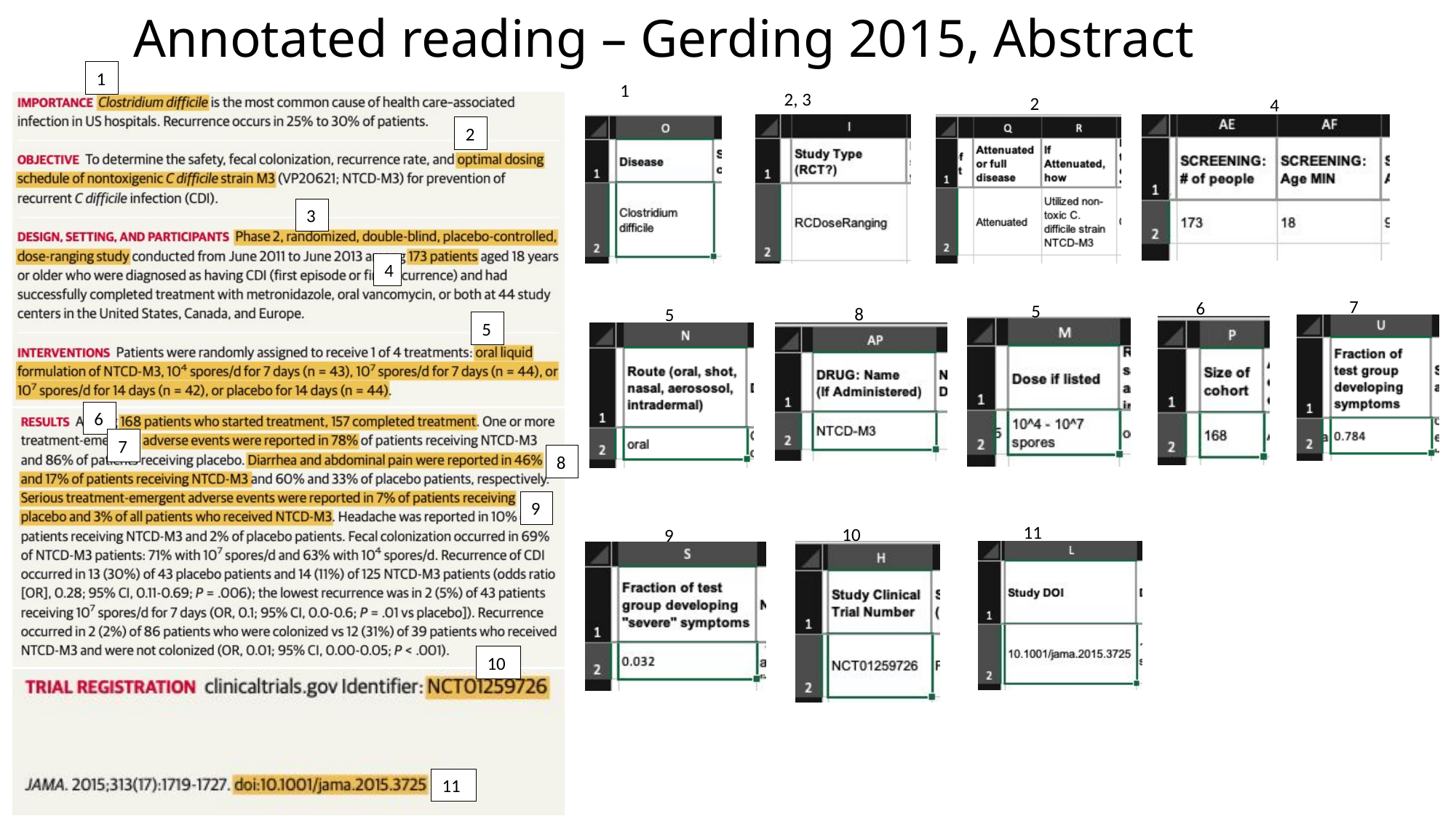

# Annotated reading – Gerding 2015, Abstract
1
1
2, 3
2
4
2
3
4
7
6
5
8
5
5
6
7
8
9
11
10
9
10
11

## Slide 8
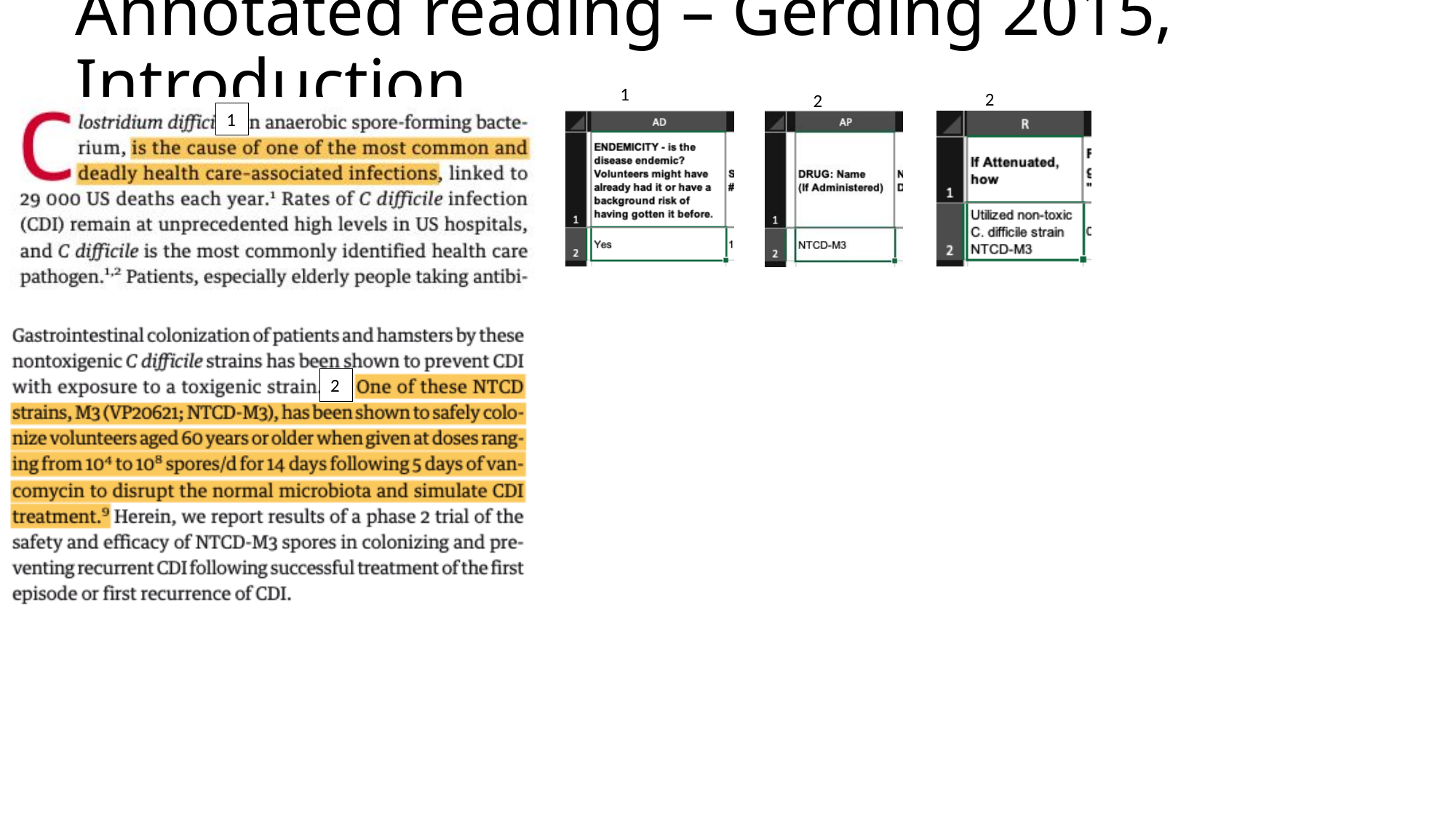

# Annotated reading – Gerding 2015, Introduction
1
2
2
1
2

## Slide 9
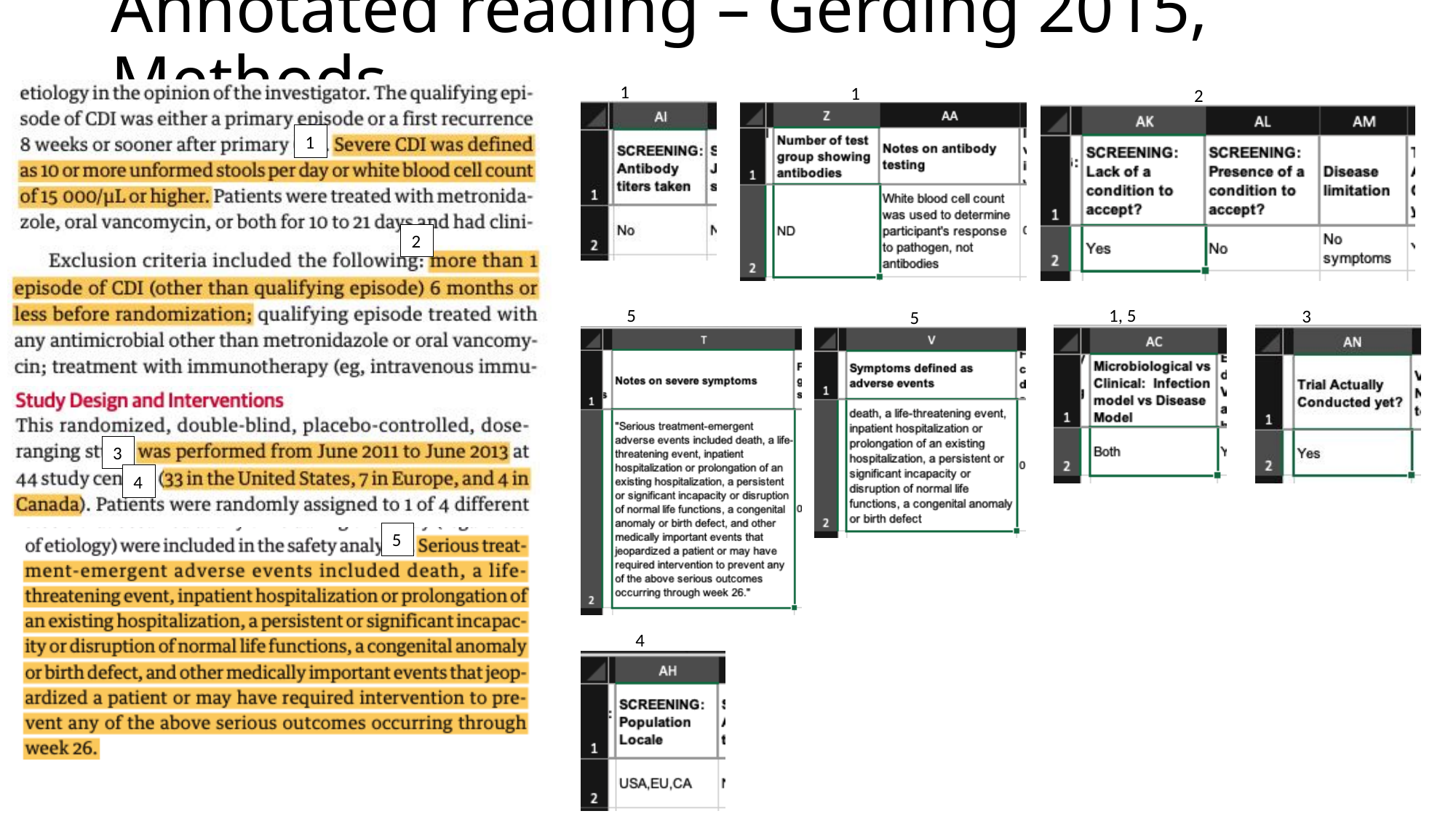

# Annotated reading – Gerding 2015, Methods
1
1
2
1
2
5
1, 5
3
5
3
4
5
4

## Slide 10
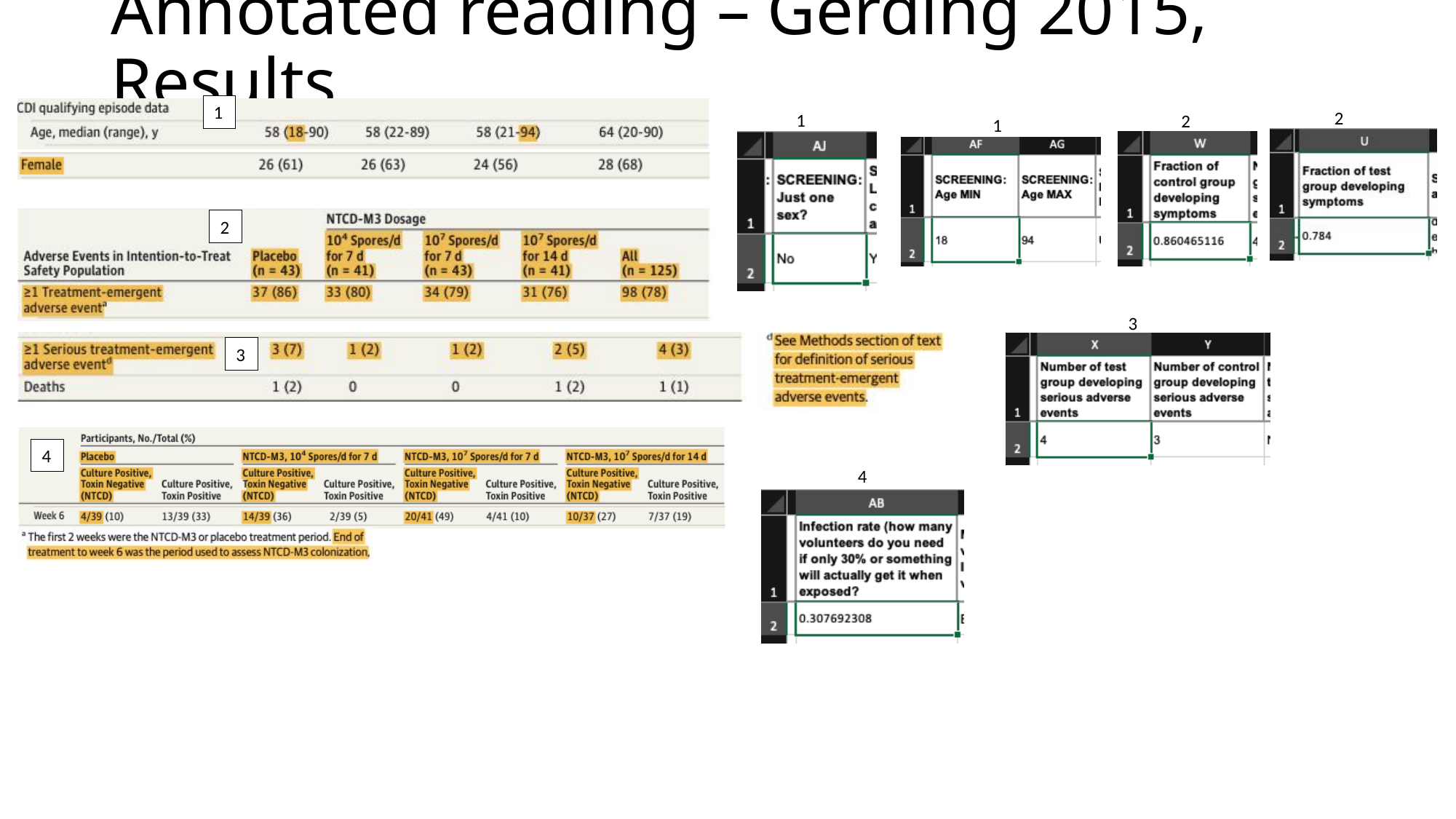

# Annotated reading – Gerding 2015, Results
1
2
1
2
1
2
3
3
4
4

## Slide 11
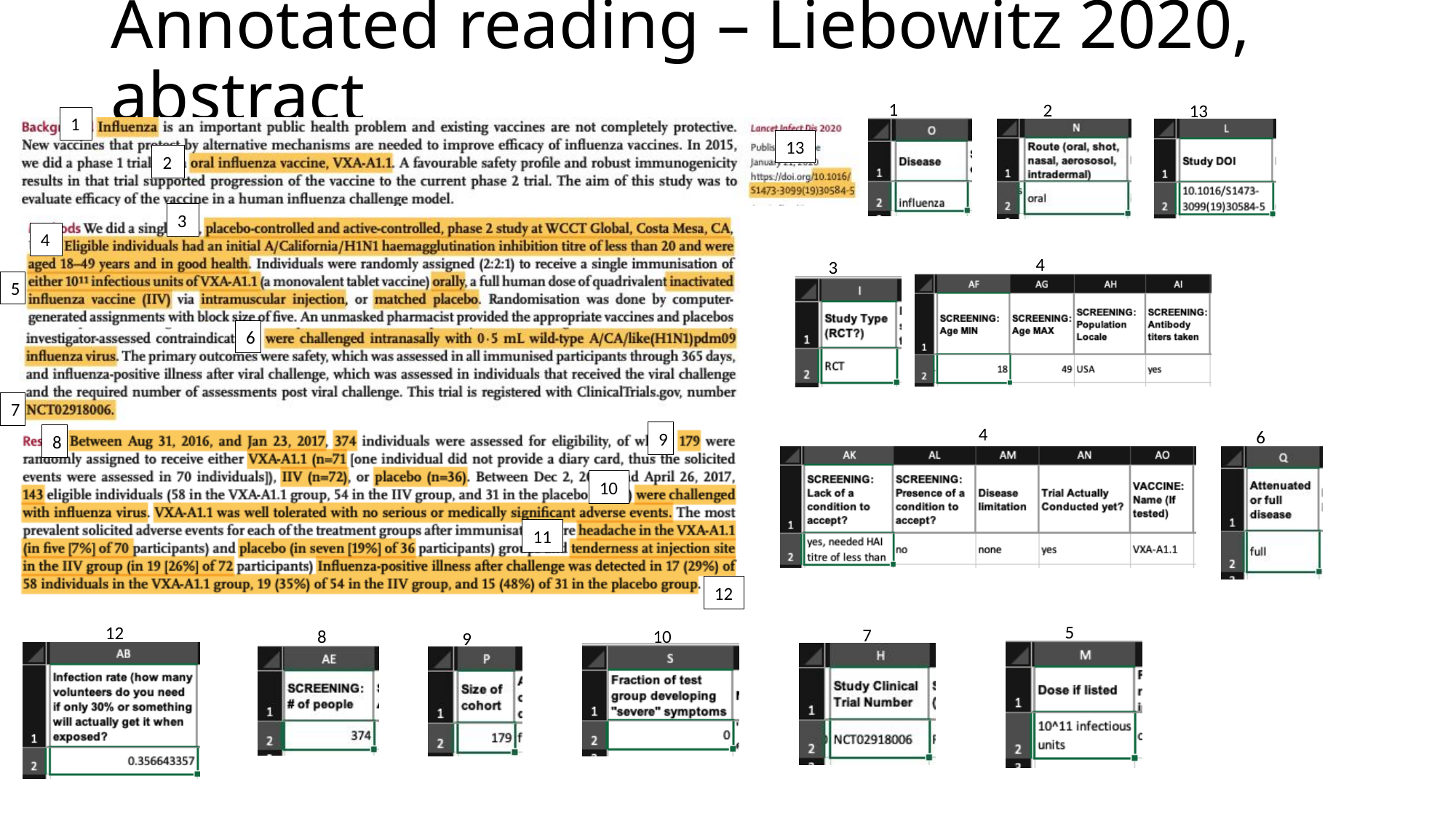

# Annotated reading – Liebowitz 2020, abstract
1
2
13
1
13
2
3
4
4
3
5
6
7
4
6
9
8
10
11
12
5
12
7
8
10
9

## Slide 12
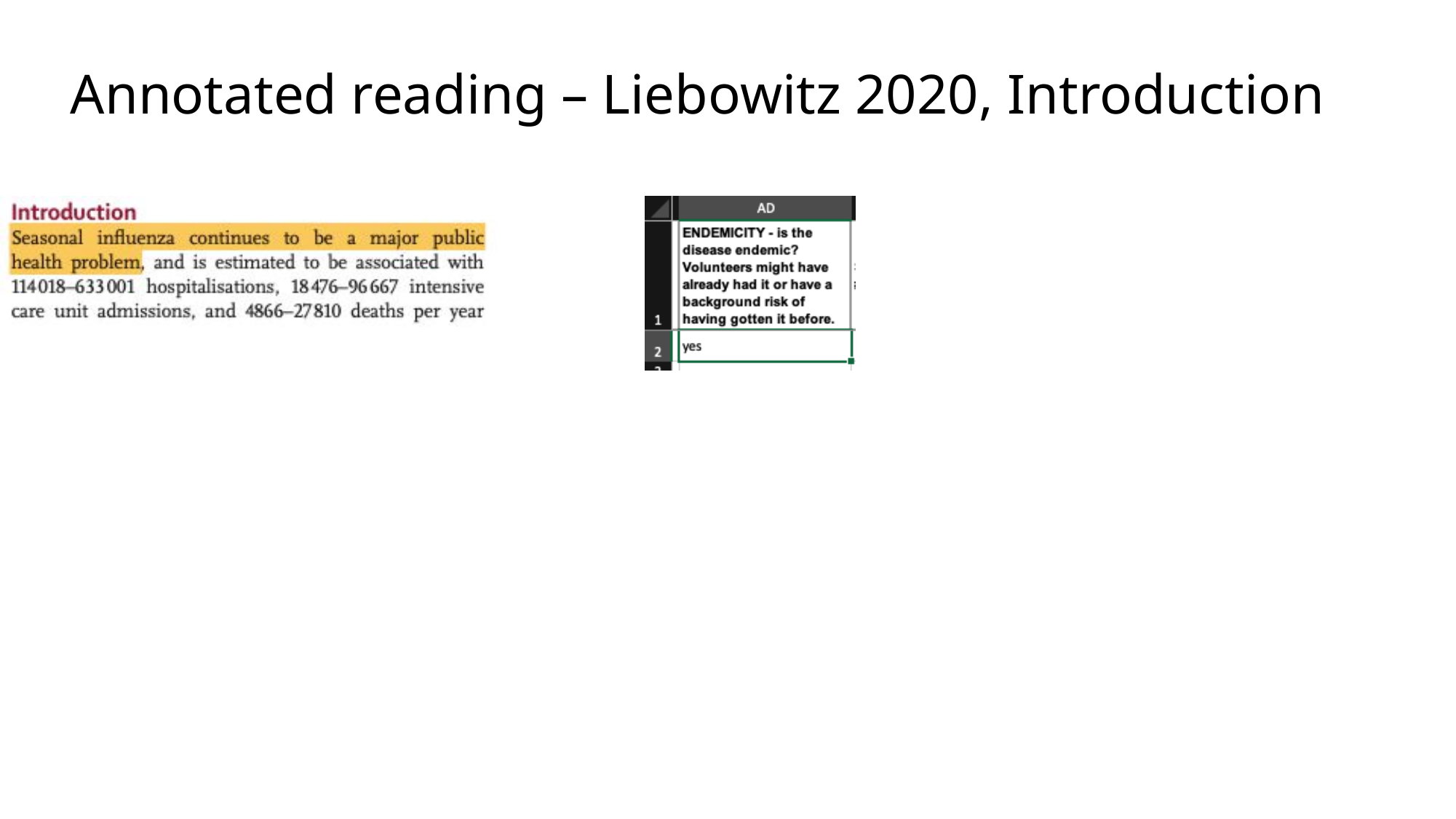

# Annotated reading – Liebowitz 2020, Introduction

## Slide 13
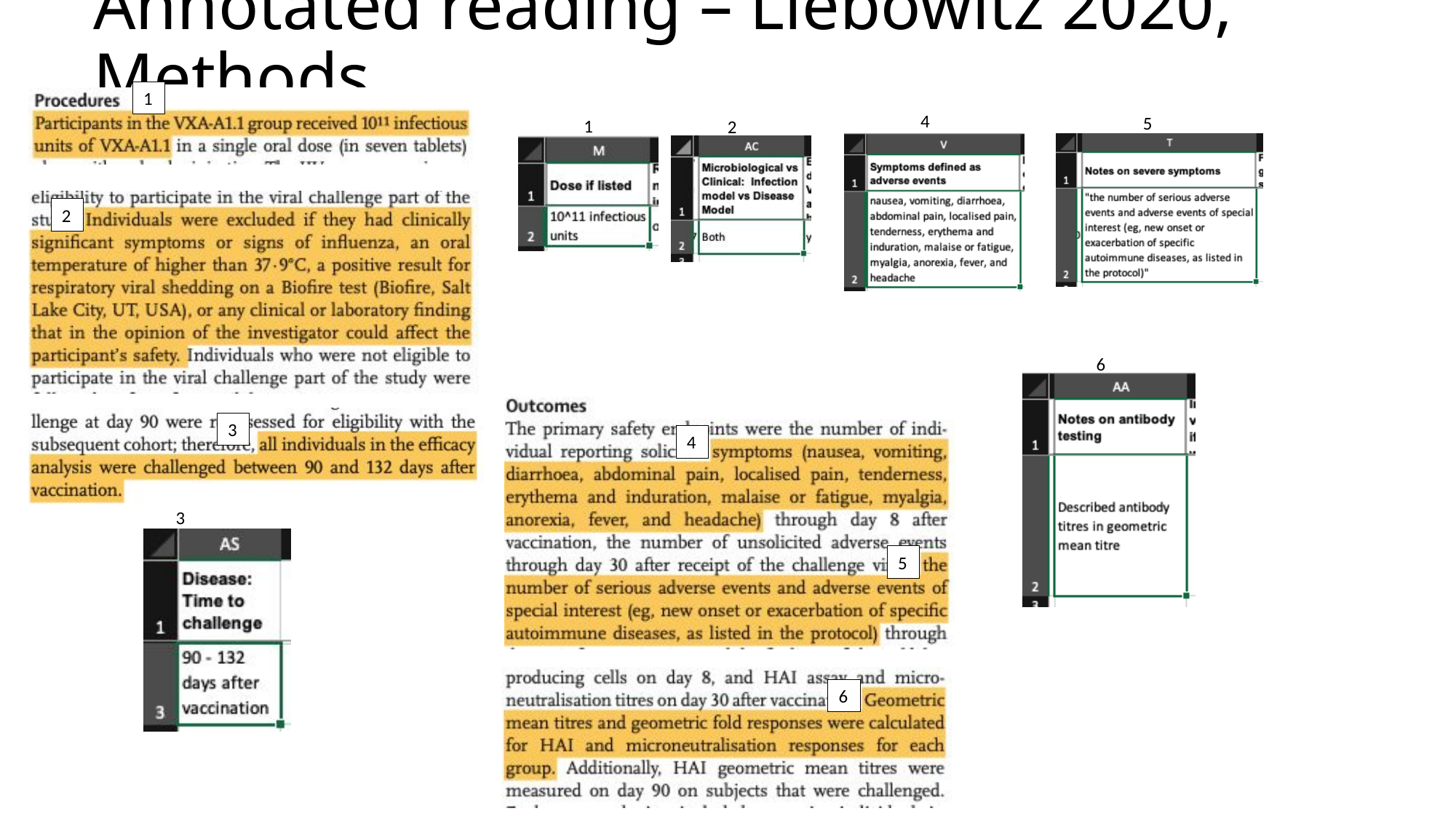

# Annotated reading – Liebowitz 2020, Methods
1
4
5
1
2
2
6
3
4
3
5
6

## Slide 14
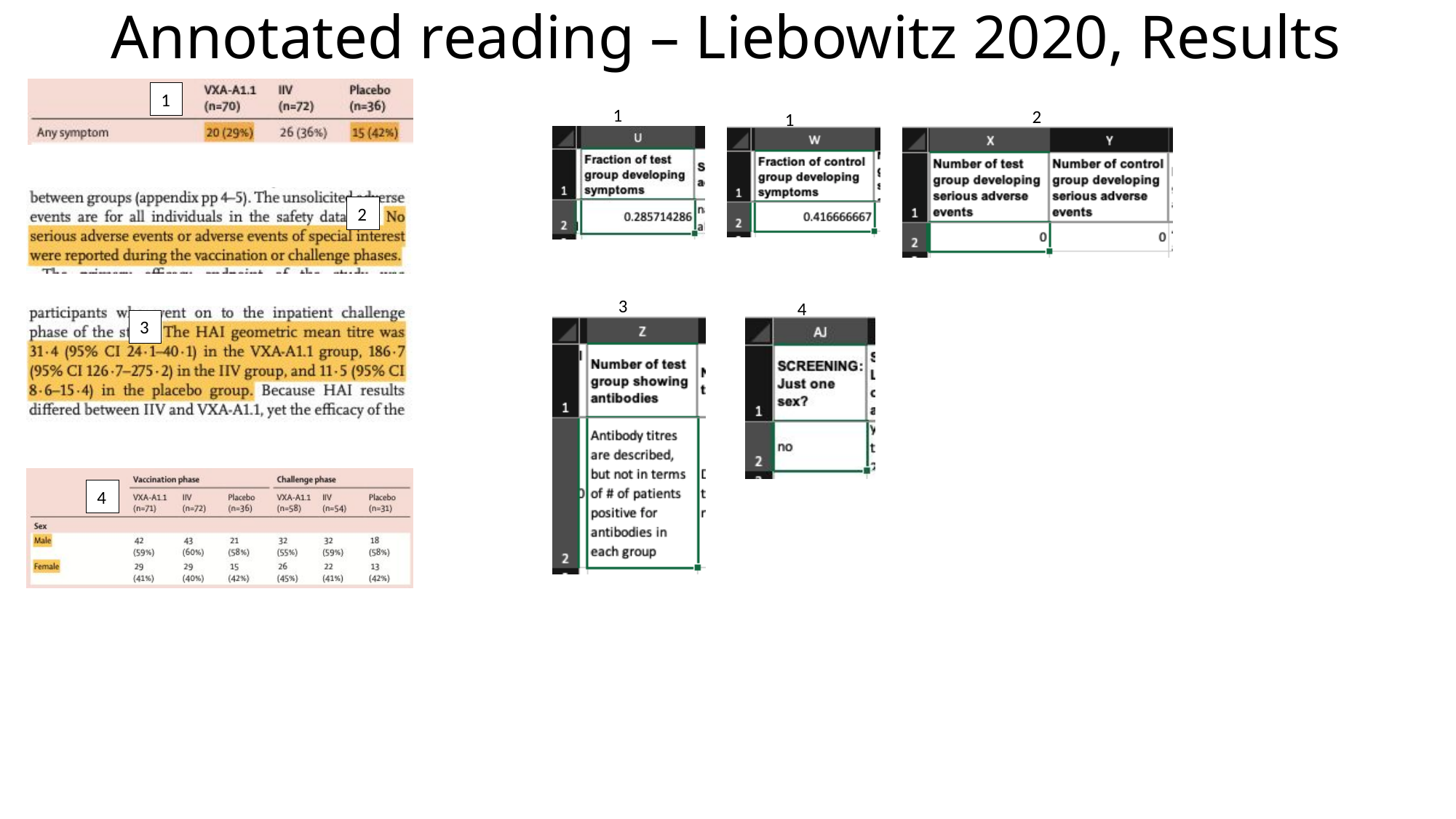

# Annotated reading – Liebowitz 2020, Results
1
1
2
1
2
3
4
3
4

## Slide 15
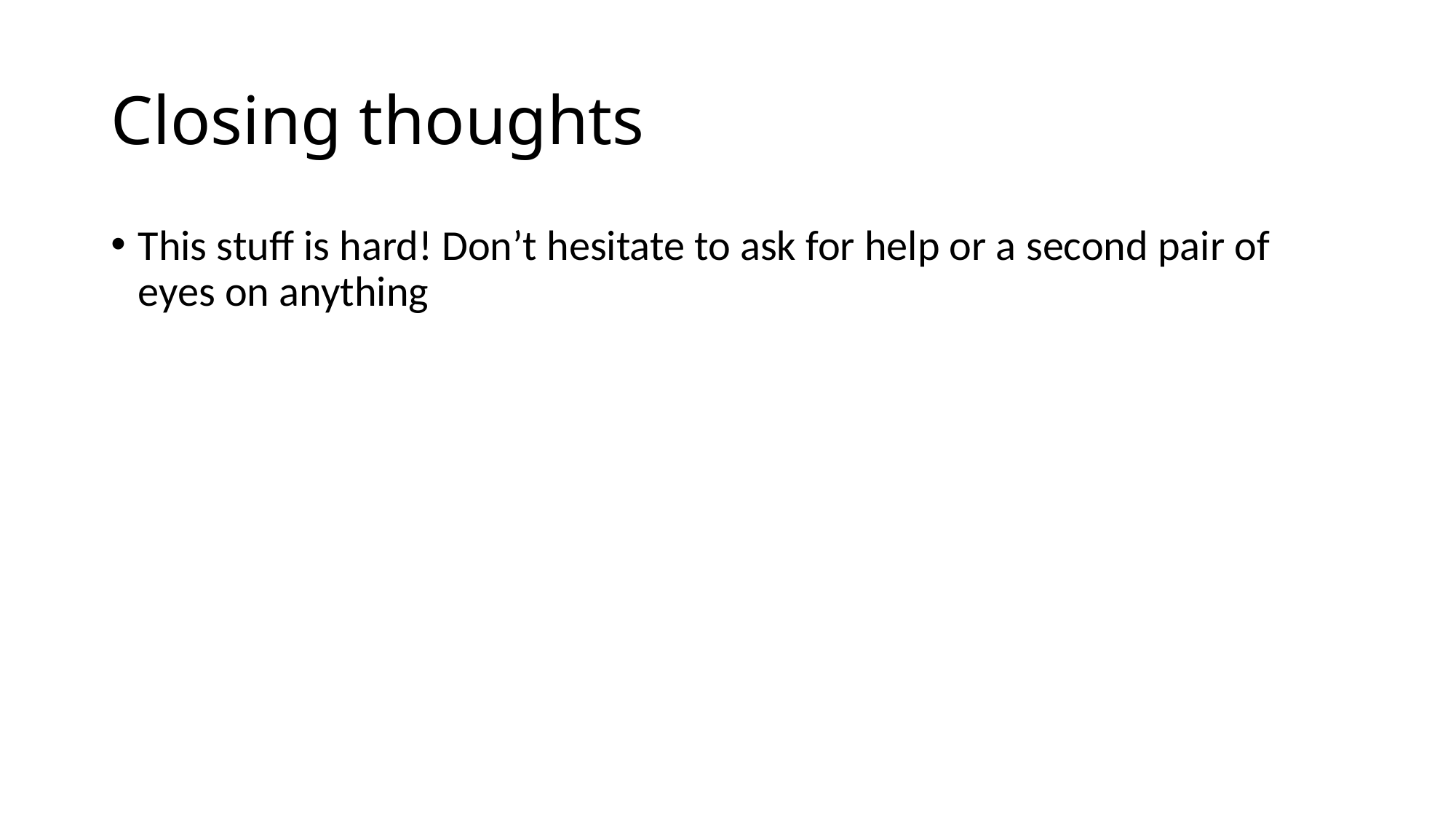

# Closing thoughts
This stuff is hard! Don’t hesitate to ask for help or a second pair of eyes on anything
